# Supplementary material for: USP7 and USP47 deubiquitinases regulate NLRP3 inflammasome activation
Source: EMBO Rep. 2018 Sep 11;19(10):e44766. doi: 10.15252/embr.201744766 (PMC6172458; doi:10.15252/embr.201744766)
Supplement: Supplementary file 1 — Appendix [file EMBR-19-e44766-s001.pdf]

APPENDIX

Table of contents:

Appendix Fig S1. USP7 and USP47 are closely related DUBs…………………………….…1

Appendix Fig S2. P5091 and HBX19818 also block inflammasome activation……………..2

Appendix Fig S3. THP-1 response to Poly (dA:dT) is NLRP3 dependent …………………..3

Appendix Fig S4. Transcriptional regulation of USP7, USP47 and BRCC3, in response to LPS in human macrophages………………………………………………………………….4

Appendix Fig S5. Inducible CRISPR/Cas9 knockout for USP7 and USP47 with different guides confirms their role in inflammasome activation………………………………………..5

Appendix Fig S6. USP7 and USP47 deficiency does not affect production of IL-6 and TNFα……………………………………………………………………………………………..6

A.

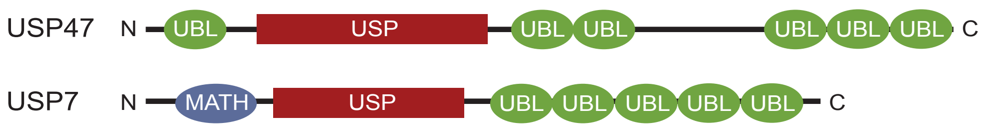

B.

```
UBP7_HUMAN          1 MNHQQQQQQQKAGEQQLSEPEDM----------EME------AGDTDDP----------------PRITQN-----PV-------------INGNVAL--     48
                              .....|.||...||:          ||:      ....::|                .|||.|     ||             |||...|
UBP47_HUMAN         1 --------MVPGEENQLVPKEDVFWRCRQNIFDEMKKKFLQIENAAEEPRVLCIIQDTTNSKTVNERITLNLPASTPVRKLFEDVANKVGYINGTFDLVW     92

UBP7_HUMAN         49 SDGHNTAEEDMEDDTSWRS--EATFQFTVERFSRLSESVLSPPCFVRNLPWKIM----------VMPRFYPDRPHQKSVGFFLQCNAESDSTSWSCHAQA    136
                      .:|.|||:....|.||.:|  :|.|:...:.|..|:.....|         :|:          |..||....|.:.|.|      :.||..|.|....:
UBP47_HUMAN        93 GNGINTADMAPLDHTSDKSLLDANFEPGKKNFLHLTDKDGEQP--------QILLEDSSAGEDSVHDRFIGPLPREGSGG------STSDYVSQSYSYSS    178

UBP7_HUMAN        137 VLKIINYRDDEKSFSRRISHLFFHKENDWGFSNFMAWSEVTDPEKGFIDDDKVTFEVFVQADAPHGVAWDSKKHTGYVGLKNQGATCYMNSLLQTLFFTN    236
                      :|                                                                    :|..||||||.||..|||:||||||||.|.
UBP47_HUMAN       179 IL--------------------------------------------------------------------NKSETGYVGLVNQAMTCYLNSLLQTLFMTP    210

UBP7_HUMAN        237 QLRKAVYMMPTE--GDDSSKSVPLALQRVFYELQHSDK-PVGTKKLTKSFGWETLDSFMQHDVQELCRVLLDNVENKMKGTCVEGTIPKLFRGKMVSYIQ    333
                      :.|.|:|....|  .:|...|:|..|||:|..||.|.| .:.|..:|:||||::.:::.||||||||||:.|.:|.|.|.|.....|.:|::||:..|::
UBP47_HUMAN       211 EFRNALYKWEFEESEEDPVTSIPYQLQRLFVLLQTSKKRAIETTDVTRSFGWDSSEAWQQHDVQELCRVMFDALEQKWKQTEQADLINELYQGKLKDYVR    310

UBP7_HUMAN        334 CKEVDYRSDRREDYYDIQLSIK--GKKNIFESFVD----YVAVEQLDGDNKYDAGEHGLQ-EAEKGVKFLTLPPVLHLQLMRFMYDPQTDQNIKINDRFE    426
                      |.|..|...|.:.|.||.|.|:  |....|.|..:    ::..|.|||.|:|.......: :|.||::||..|.:|.|||.||.:|..|...||:|||..
UBP47_HUMAN       311 CLECGYEGWRIDTYLDIPLVIRPYGSSQAFASVEEALHAFIQPEILDGPNQYFCERCKKKCDARKGLRFLHFPYLLTLQLKRFDFDYTTMHRIKLNDRMT    410

UBP7_HUMAN        427 FPEQLPLDEFLQKTDPKDPAN-------------------------------------------------------YILHAVLVHSGDNHGGHYVVYLNP    471
                      |||:|.:..|:...|.|.|..                                                       |.|.:|:||||...||||...:..
UBP47_HUMAN       411 FPEELDMSTFIDVEDEKSPQTESCTDSGAENEGSCHSDQMSNDFSNDDGVDEGICLETNSGTEKISKSGLEKNSLIYELFSVMVHSGSAAGGHYYACIKS    510

UBP7_HUMAN        472 KGDGKWCKFDDDVVSRCTKEEAIEHNYGGHDD-----DLSVRHCTNAYMLVY-----IRESKLSEVLQAVTDHDIPQQLVERLQEEKRIEAQKRKERQEA    561
                      ..|.:|..|:|..|||.|:|: |:..:||...     ..:....||||||:|     .|.:|..||      .:.|:.:...:|:|:.:|.|::::|:..
UBP47_HUMAN       511 FSDEQWYSFNDQHVSRITQED-IKKTHGGSSGSRGYYSSAFASSTNAYMLIYRLKDPARNAKFLEV------DEYPEHIKNLVQKERELEEQEKRQREIE    603

UBP7_HUMAN        562 H---------LYMQVQIVAEDQFCGHQGNDM--------------------------YDE-----EK----------------VKYT-------------    592
                      .         |:...|::.|::...|:...:                          |||     |:                ||.|
UBP47_HUMAN       604 RNTCKIKLFCLHPTKQVMMENKLEVHKDKTLKEAVEMAYKMMDLEEVIPLDCCRLVKYDEFHDYLERSYEGEEDTPMGLLLGGVKSTYMFDLLLETRKPD    703

UBP7_HUMAN        593 -VFKVLK----------------------------NSSLAEFVQSLSQTMGFPQDQIRLWPMQARSNGTKRPAMLDN---EADG----NKTMIELSDNEN    656
                       ||:..|                            |.::.||.|.:|:.:..|.:.:|: :..|.....|...:.:   :|:|    ||..:|.|
UBP47_HUMAN       704 QVFQSYKPGEVMVKVHVVDLKAESVAAPITVRAYLNQTVTEFKQLISKAIHLPAETMRI--VLERCYNDLRLLSVSSKTLKAEGFFRSNKVFVESS----    797

UBP7_HUMAN        657 PWTIFLETVDPELAASGATLPKFDKDH--DVMLFLKMYDPKTRSLNY-------------------CGHIYTPISCKIRDLLPVM---------------    720
                            ||:|.::|.:.:.|.|....|  .:.||:.:  |:...::|                   |..:..|:. .::.:..::
UBP47_HUMAN       798 ------ETLDYQMAFADSHLWKLLDRHANTIRLFVLL--PEQSPVSYSKRTAYQKAGGDSGNVDDDCERVKGPVG-SLKSVEAILEESTEKLKSLSLQQQ    888

UBP7_HUMAN        721 --CDRAGFIQDTSLILYEEVKPNLTERIQDYDVSLD-KALDELMDGDIIVFQKDDPENDNSELPTAKEYFRD------------LYHRVDVIFCDKTIPN    805
                        .|.....:.|....:|.::..|.||  |...|:| :.|::.:       |..||||..||..:.:...|            ..|..|      |:.|
UBP47_HUMAN       889 QDGDNGDSSKSTETSDFENIESPLNER--DSSASVDNRELEQHI-------QTSDPENFQSEERSDSDVNNDRSTSSVDSDILSSSHSSD------TLCN    973

UBP7_HUMAN        806 DPGFVVTLSNRMNYFQVAKTVAQRLN---------------TD-----------PMLLQFFKSQGYRDGPGNPLRHNYEGTLRDLLQFFKPRQPKKLYYQ    879
                      .....:.|:|.::...:.:...:.|               ||           .|...:||::.|....|:...|.:      |:.....|.....:.|
UBP47_HUMAN       974 ADNAQIPLANGLDSHSITSSRRTKANEGKKETWDTAEEDSGTDSEYDESGKSRGEMQYMYFKAEPYAADEGSGEGHKW------LMVHVDKRITLAAFKQ   1067

UBP7_HUMAN        880 QLK---------MKI-TDFENRRSFKCIWLN---SQFREE-EITL----------YPDKHGCVRDLL----EECKKAVELGEKASGKLRLLEIVSYKIIG    951
                      .|:         .|: ..:.:.:.|:.:.||   |.|.:: :||:          |..|    |..||    |.||                .||:.|..|.:.
UBP47_HUMAN      1068 HLEPFVGVLSSHFKVFRVYASNQEFESVRLNETLSSFSDDNKITIRLGRALKKGEYRVK---VYQLLVNEQEPCK------------FLLDAVFAKGMT   1151

UBP7_HUMAN        952 VHQEDELL------ECLSPATSRTFRI-------------------EEI-----------PLDQVDIDKENEMLVTVAHFHKEVFGTFGIPFLLRIHQGE   1015
                      |.|..|.|      :|....:...||:                   |:|           .||.|:..|....|..::...|          |..:::   :
UBP47_HUMAN      1152 VRQSKEELIPQLREQCGLELSIDRFRLRKKTWKNPGTVFLDYHIYEEDINISSNWEVFLEVLDGVEKMKSMSQLAVLSRRWK--------PSEMKL---D   1240

UBP7_HUMAN       1016 HFREVMKRIQSLLDIQEK------------EFEK----FKFAIVMMGRHQ----------------YINEDEYEVNLKDFEPQPGNMSHPRPWLGLDHFN   1083
                      .|:||:....|:.:::||            ||.|    |...|.::..||                ||.:|...:..:|...:...::.        :..|
UBP47_HUMAN      1241 PFQEVVLESSSVDELREKLSEISGIPLDDIEFAKGRGTFPCDISVLDIHQDLDWNPKVSTLNVWPLYICDDGAVIFYRDKTEELMELTD-------EQRN   1333

UBP7_HUMAN       1084 KAPKRS---------RYTY---LEKAIKIHN-----------   1102
                      :..|:.         |.||   .|||:||:.
UBP47_HUMAN      1334 ELMKKESSRLQKTGHRVTYSPRKEKALKIYLDGAPNKDLTQD   1375
```

Appendix Figure S1. USP7 and USP47 are closely related DUBs.

A. USP7 and USP47 present a high similarity in domain structure. Domains shown represent: UBL (Ubiquitin-like domain; green), MATH domain (blue) and USP domain (red). The N- and C-terminal ends are labelled.

B. USP7 (Q93009) and USP47 (Q96K76) pairwise protein sequence alignment using EMBOSS needle (https://www.ebi.ac.uk/Tools/psa/emboss_needle/). The two catalytic sites share 35.2% identity (denoted with ‘|’; 133/378) and 48.4% similarity (denoted with ‘.’ or ‘:’; 183/378). Domains are labelled as in A.

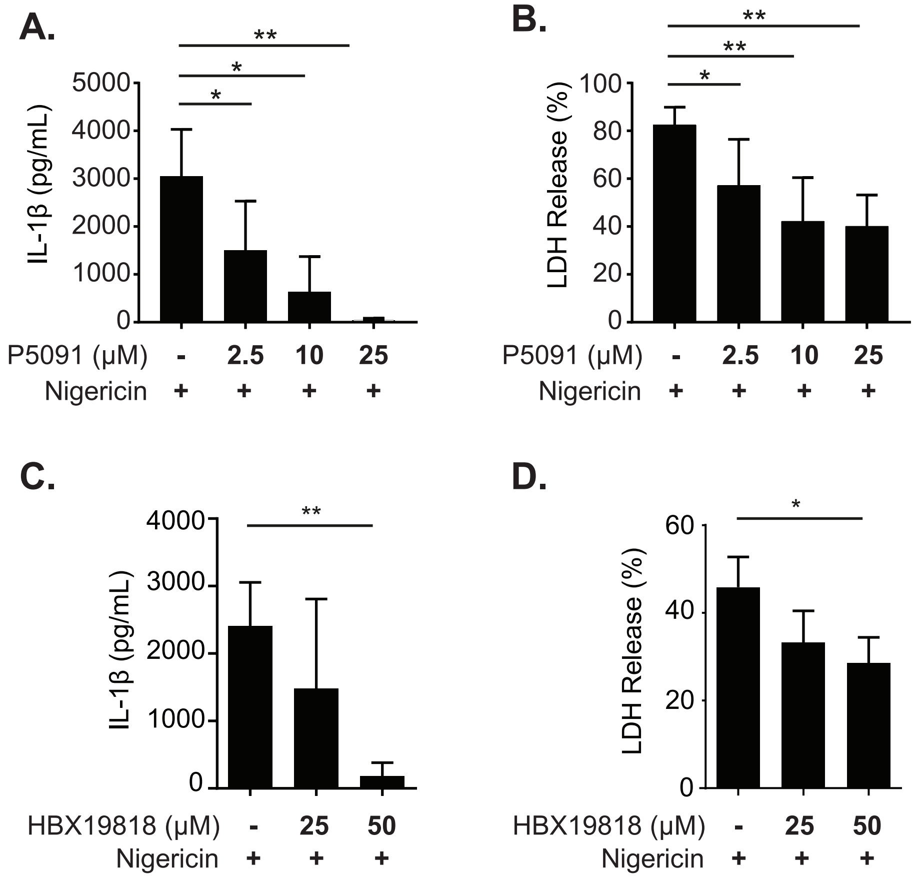

Appendix Figure S2. P5091 and HBX19818 also block inflammasome activation.

A. IL-1β ELISA in supernatants of LPS-primed (1 μg/mL, 4 hrs.) MDMs pre-incubated with either DMSO or P5091 at the indicated concentrations for 15 mins. before treatment with nigericin (10 μM, 45 mins.). Bars represent the mean ± S.D. n = 4 independent blood donors. * = $P < 0.05$ and ** = $P < 0.01$ using a t-test between each condition and nigericin alone.

B. LDH release in supernatants of MDMs as treated in A. Bars represent the mean ± S.D. n = 4 independent blood donors. * = $P < 0.05$ and ** = $P < 0.01$ using a t-test between each condition and nigericin alone.

C. IL-1β ELISA in supernatants of LPS-primed (1 μg/mL, 4 hrs.) MDMs pre-incubated with either DMSO or HBX19818 at the indicated concentrations for 15 mins. before treatment with nigericin (10 μM, 45 mins.). Bars represent the mean ± S.D. n = 4 independent blood donors. ** = $P < 0.01$ using a t-test between each condition and nigericin alone.

D. LDH release in supernatants of MDMs as treated in C. Bars represent the mean ± S.D. n = 4 independent blood donors. * = $P < 0.05$ using a t-test between each condition and nigericin alone.

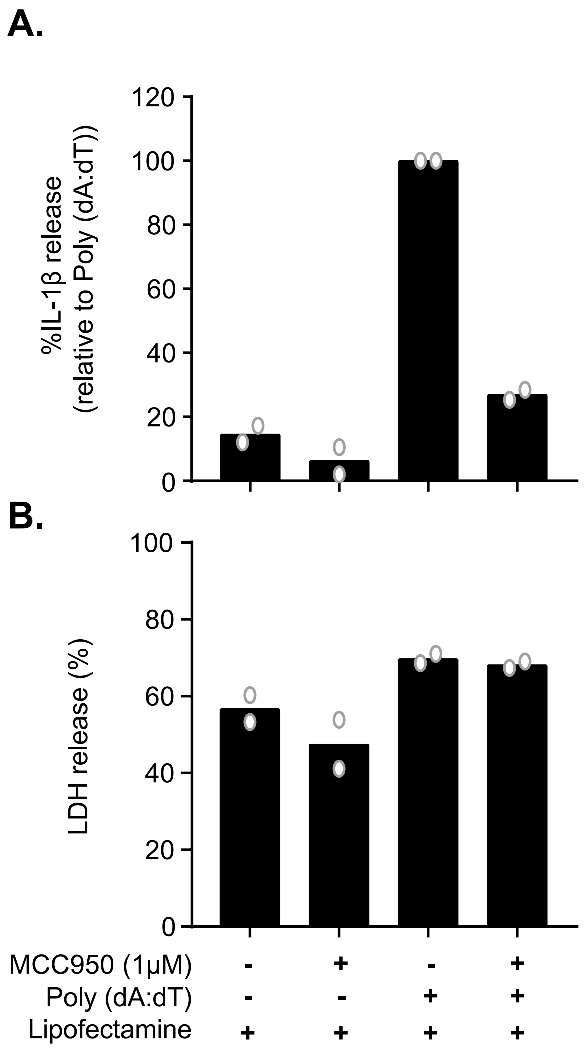

Appendix Figure S3. THP-1 response to Poly(dA:dT) is NLRP3 dependent.
A. IL-1β ELISA of supernatants from LPS-primed (1 μg/mL, 4 hrs.) PMA-diffrentiated THP1 cells pre-incubated with either 0.1% DMSO or MCC950 (1 μM) 15 mins. before treatment with Poly (dA:dT) (1 μg/mL; 24 hrs.), as indicated. Data representated as the percentage of IL-1β release relative to the release after Poly (dA:dT) treatment. Bars represent the mean of two independent biological replicates. n =2
B. LDH-release from THP1 cells treated as in A.

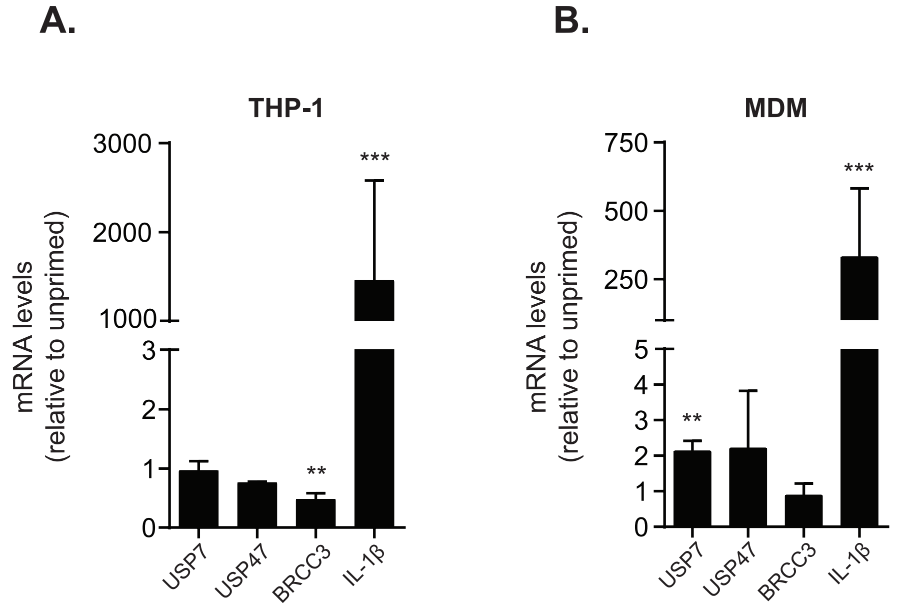

Appendix Figure S4. Transcriptional regulation of USP7, USP47 and BRCC3, in response to LPS in human macrophages.

A. Quantitative real-time (qRT)-PCR for USP7, USP47, BRCC3 and IL-1β gene expression in response to LPS priming (1 µg/mL, 4hrs.) in PMA-differentiated THP-1 cells. Bars represent the mean expression level, relative to untreated cells, ± S.D. n = 3 independent biological experiments. ** = P < 0.01 and *** = P < 0.001 using a t-test analysis comparing each condition to the untreated control.

B. Quantitative real-time (qRT)-PCR for the genes as in A, but in MDMs. Bars represent the mean expression level, relative to untreated cells, ± S.D. n = 3 independent human blood donors. ** = P < 0.01 and *** = P < 0.001 using a t-test analysis comparing each condition to the untreated control.

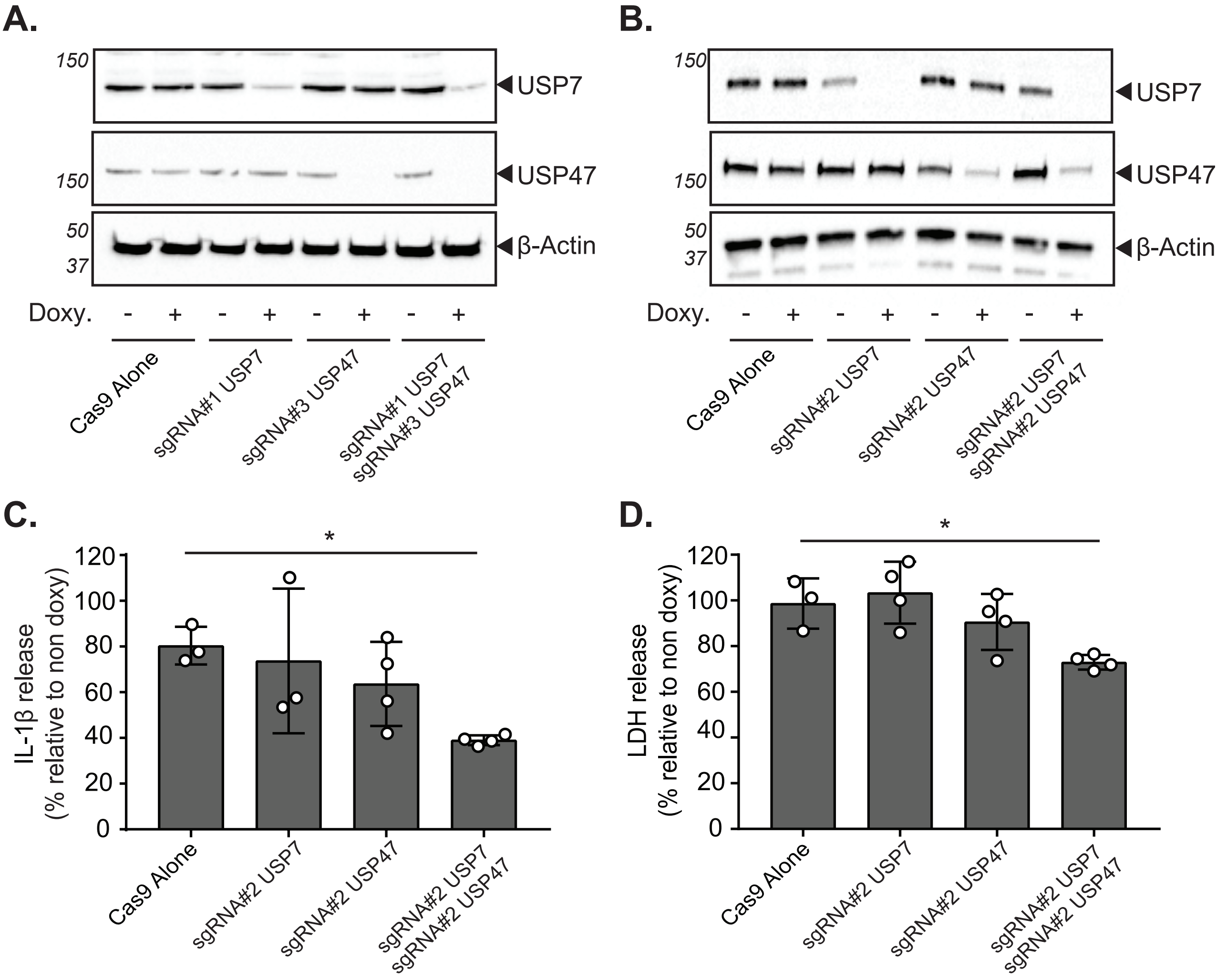

Appendix Figure S5. Inducible CRISPR/Cas9 knockout for USP7 and USP47 with different guides confirms their role in inflammasome activation.

A. Western blots of cell lysates from PMA-differentiated THP-1 cells engineered by CRISPR/Cas9 to induce USP7 and/or USP47 KOs. Deficiency of both USP7 and USP47 was induced by doxycycline (Doxy) treatment (1 µg/mL; 3 days), with the indicated sgRNA (sgRNA#1 USP7 and sgRNA#3 USP47). Bands in the figure represent: USP7, USP47 and β-actin (as a loading control). Blots are representative of at least 3 independent experiments.

B. Western blot of cell lysates from PMA-differentiated THP-1 cells engineered by CRISPR/Cas9 to induce USP7 and/or USP47 KOs using a different set of sgRNAs (sgRNA#2 USP7 and sgRNA#2 USP47). Blots are representative of at least 3 independent experiments.

C. IL-1β release measured from doxycycline treated (1 µg/mL; 3 days) PMA differentiated and LPS-primed (1 µg/mL, 4 hrs.), nigericin activated (10 µM, 45 mins) THP-1 cells, which contain the indicated sgRNA. Bars represent the mean percentage of IL-1β release relative to their respective non-doxycycline treated control cells, ± S.D. n = 3-4 (as indicated) independent biological replicates, plotted as open circles on each bar. * = $P < 0.05$ using a one-way ANOVA.

D. LDH-release measured from cells as treated in B. Bars represent the mean percentage of LDH release relative to their respective non-doxycycline treated control cells, ± S.D. n = 3-4 (as indicated) independent biological replicates, plotted as open circles on each bar. * = $P < 0.05$ using a one-way ANOVA.

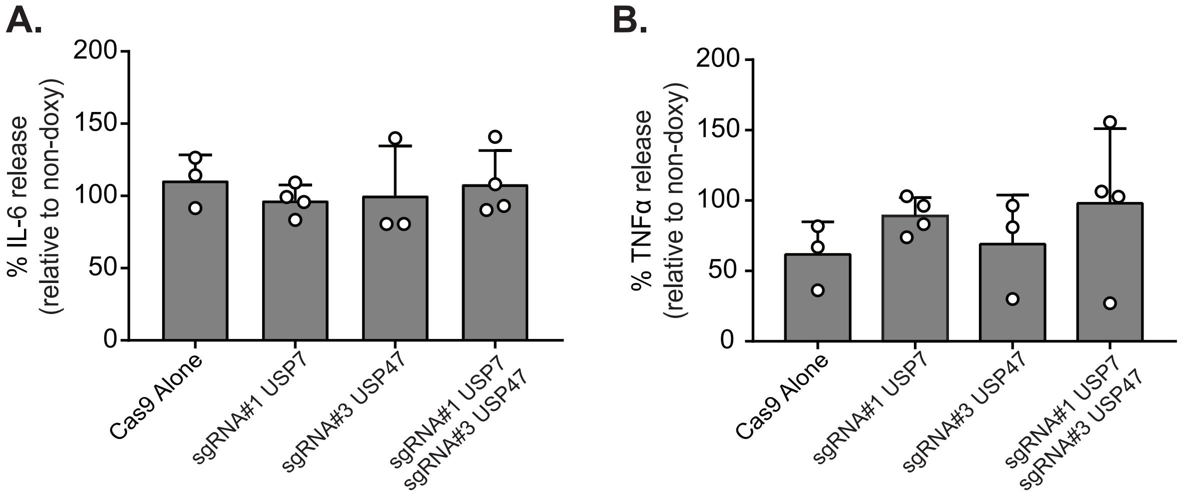

Appendix Figure S6. USP7 and USP47 deficiency does not affect production of IL-6 and TNFα.

A. IL-6 ELISA in supernatants of PMA differentiated and LPS-primed (1 µg/mL, 4 hrs.) THP-1 cells engineered by CRISPR/Cas9 to induce USP7 and/or USP47 KOs. Deficiency of both USP7 and USP47 was induced by doxycycline (Doxy) treatment (1 µg/mL; 3 days), with the indicated sgRNA. Data are presented as the mean percentage of cytokine release, relative to their non-doxycycline treated control, ± S.D. n = 3-4 (as indicated) independent biological replicates, plotted as open circles on each bar. No statistical difference was found using a one-way ANOVA.

B. TNFα ELISA in supernatants of cells treated and analysed as in A.
