# Supplementary material for: USP7 and USP47 deubiquitinases regulate NLRP3 inflammasome activation
Source: EMBO Rep. 2018 Sep 11;19(10):e44766. doi: 10.15252/embr.201744766 (PMC6172458; doi:10.15252/embr.201744766)
Supplement: Supplementary file 2 — Expanded View Figures PDF [file EMBR-19-e44766-s002.pdf]

Expanded View Figures

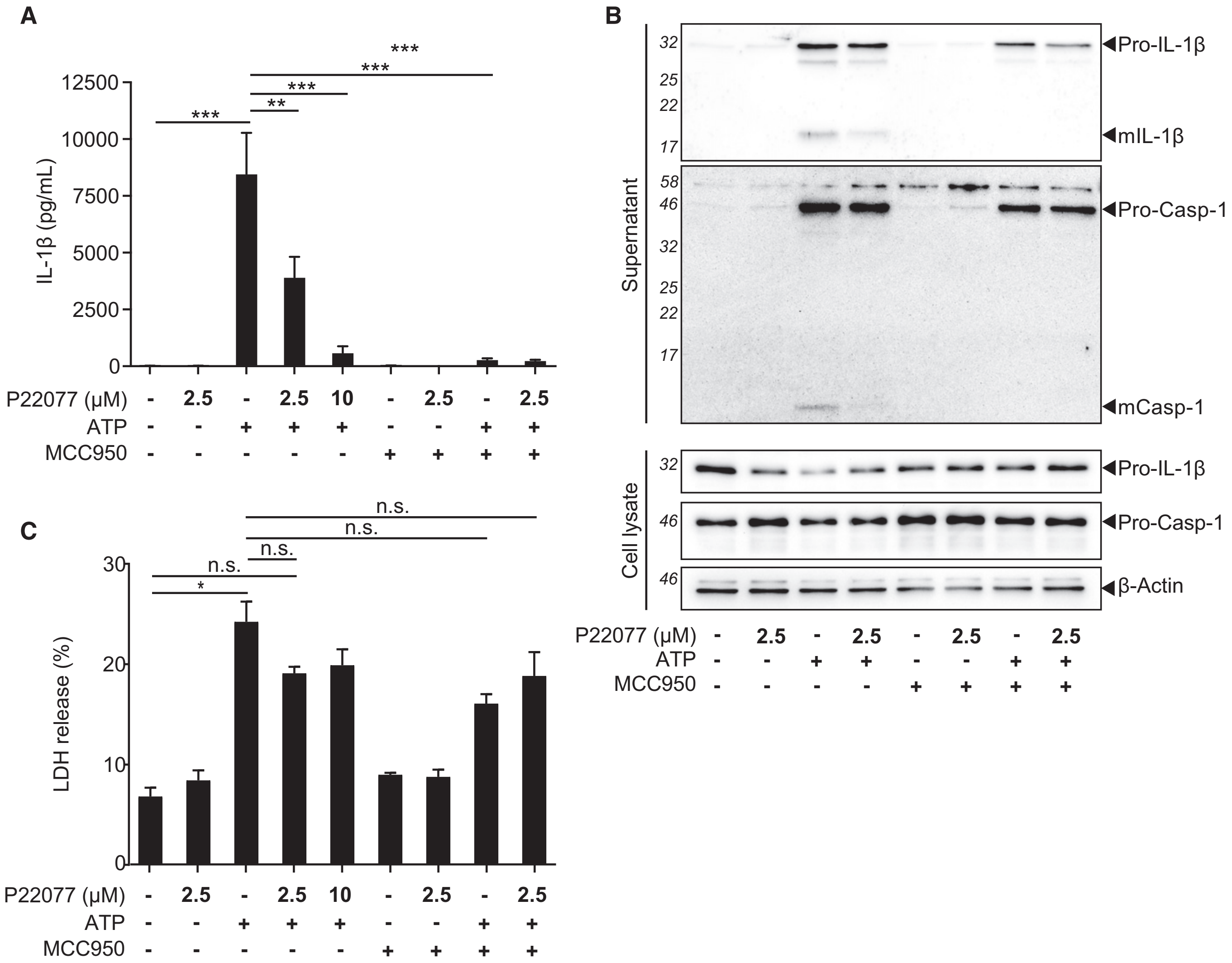

Figure EV1. Inhibition of USP7 and USP47 blocks NLRP3 inflammasome activation in mouse BMDMs.

A IL-1β ELISA in supernatants of LPS-primed (1 μg/ml, 4 h) murine BMDMs pre-incubated with either 0.1% DMSO, P22077 (at the indicated concentrations) or MCC950 (1 μM) for 15 min before treatment with ATP (5 mM, 1 h). Bars represent the mean ± SD, *n* = 4 independent murine donors. $^{**}P < 0.01$ and $^{***}P < 0.001$ using a one-way ANOVA.

B Western blots of supernatants and cell lysates from murine BMDMs as treated in (A). Data are representative of two independent murine donors. Bands in the figure represent the following: pro-IL-1β; mature IL-1β (mIL-1β); pro-caspase-1 (pro-Casp-1); and mature caspase-1 (mCasp-1). β-Actin is shown as a loading control.

C LDH release measured in supernatants of murine BMDMs as treated in (A). Bars represent the mean ± SD, *n* = 4 independent murine donors. $^{*}P < 0.05$ using a one-way ANOVA. n.s. = not significant.

Source data are available online for this figure.

 © 2018 The Authors

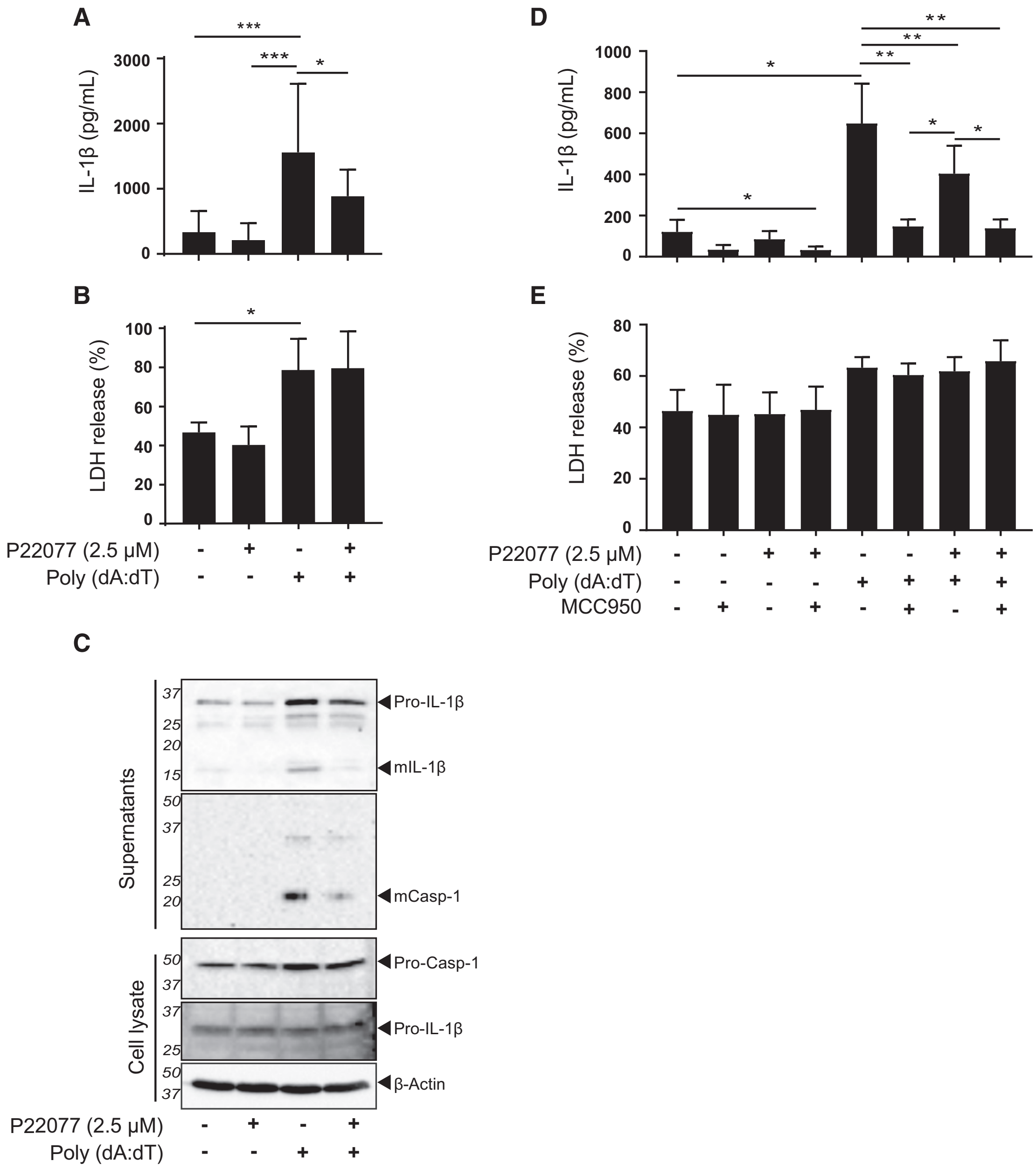

Figure EV2. Inhibition of USP7 and USP47 impairs cGAS-STING-mediated NLRP3 inflammasome activation in human macrophages.

A IL-1β ELISA of supernatants from LPS-primed (1 μg/ml, 4 h) MDMs pre-incubated with either 0.1% DMSO or P22077 (2.5 μM) 15 min before treatment with Poly (dA:dT) (1 μg/ml; 24 h), as indicated. Bars represent the mean ± SD, $n = 8$ independent blood donors. *$P < 0.05$ and ***$P < 0.001$ using a one-way ANOVA.

B LDH release from MDMs treated as in (A). Bars represent the mean ± SD, $n = 5$ independent blood donors. *$P < 0.05$ using a one-way ANOVA.

C Western blots of supernatants and cell lysates from MDMs treated as in (A). Bands in the figure represent the following: pro-IL-1β (31 kDa); mature IL-1β (mIL-1β, 17 kDa); pro-caspase-1 (pro-Casp-1, 45 kDa); and mature caspase-1 (mCasp-1, 20 kDa). β-Actin is shown as a loading control. Blots are representative of at least three independent blood donors.

D IL-1β ELISA of supernatants from LPS-primed (1 μg/ml, 4 h) MDMs pre-incubated with either 0.1% DMSO, P22077 (2.5 μM) or MCC950 (1 μM) 15 min before treatment with Poly (dA:dT) (1 μg/ml; 24 h), as indicated. Bars represent the mean ± SD, $n = 6$ independent blood donors. *$P < 0.05$ and **$P < 0.01$ using a one-way ANOVA.

E LDH release from MDMs treated as in (D). Bars represent the mean ± SD, $n = 6$ independent blood donors. No statistical differences were found using one-way ANOVA.

Source data are available online for this figure.

© 2018 The Authors

Figure EV3. Higher concentrations of USP7 and USP47 inhibitors are required to block AIM2 and NLRC4 inflammasome activation in mouse BMDMs.

A IL-1β ELISA in supernatants of LPS-primed (1 μg/ml, 4 h) murine BMDMs pre-incubated with either 0.1% DMSO (vehicle), P22077 (2.5 μM and 10 μM, as indicated) or MCC950 (1 μM) for 15 min before flagellin (667 ng/ml, 4 h) or Poly (dA:dT) (667 ng/ml, 4 h) transfection with Lipofectamine. Bars represent the mean ± SD, $n = 3$ independent murine donors. *$P < 0.05$; **$P < 0.01$ and ***$P < 0.001$ using a two-way ANOVA.

B LDH release from cells treated as in (A). Bars represent the mean ± SD, $n = 3$ independent murine donors. No statistical differences were found using two-way ANOVA.

C Western blots of supernatants and cell lysates from murine BMDMs treated with P22077 (10 μM) and Poly (dA:dT), as in (B). Data are representative of two independent murine donors. Bands in the figure represent the following: pro-IL-1β; mature IL-1β (mIL-1β); pro-caspase-1 (pro-Casp-1); and mature caspase-1 (mCasp-1). β-Actin is shown as a loading control.

D Western blots of supernatants and cell lysates from murine BMDMs treated with P22077 (10 μM) and flagellin, as in (A). Data are representative of two independent murine donors. Bands in the figure represent the same proteins as in (C).

Source data are available online for this figure.

© 2018 The Authors

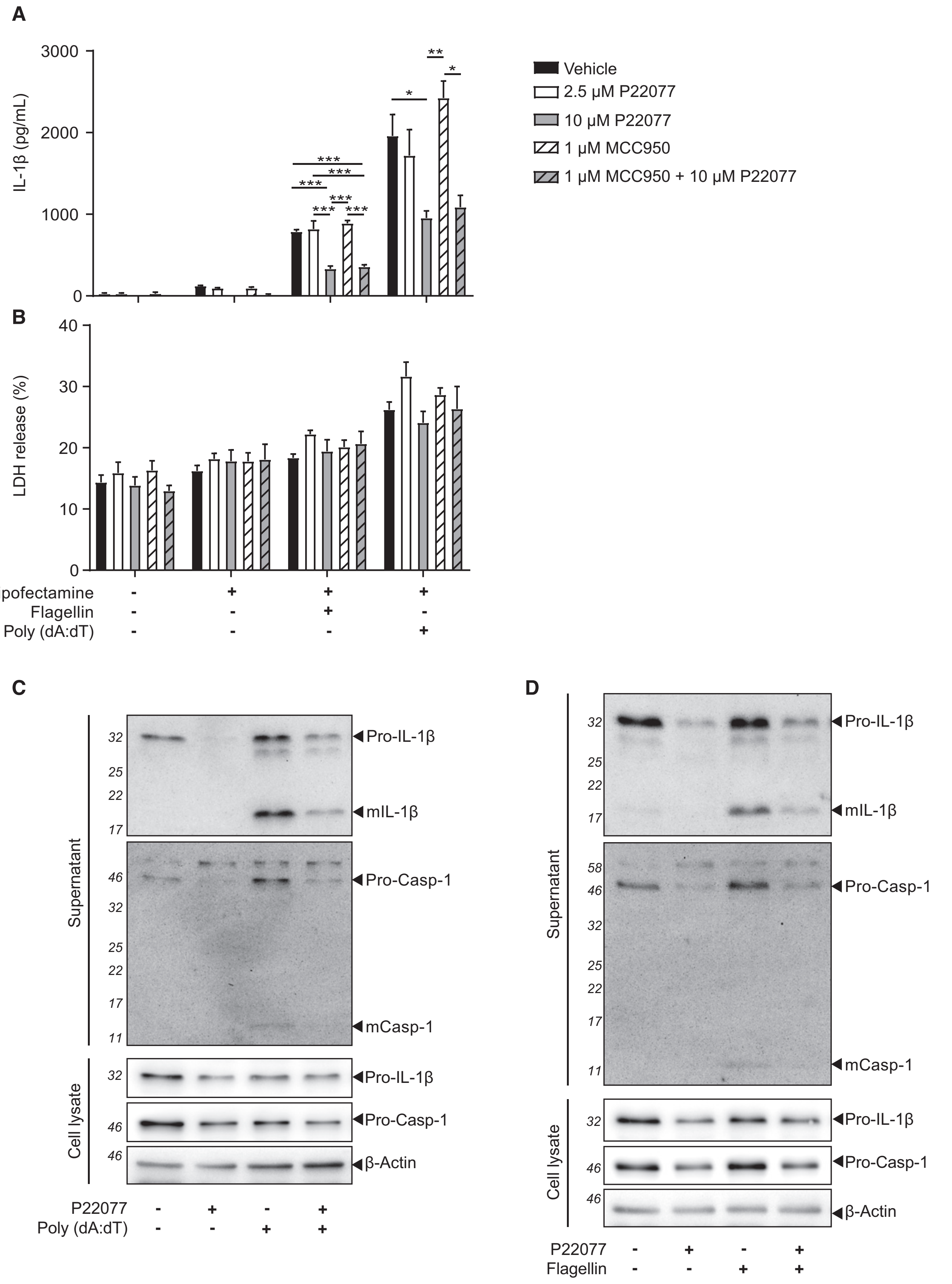
A
IL-1β (pg/mL)
Vehicle
2.5 μM P22077
10 μM P22077
1 μM MCC950
1 μM MCC950 + 10 μM P22077
B
LDH release (%)
Lipofectamine
Flagellin
Poly (dA:dT)
C
Supernatant
Cell lysate
Pro-IL-1β
mIL-1β
Pro-Casp-1
mCasp-1
β-Actin
P22077
Poly (dA:dT)
D
Supernatant
Cell lysate
Pro-IL-1β
mIL-1β
Pro-Casp-1
mCasp-1
β-Actin
P22077
Flagellin

Figure EV3.

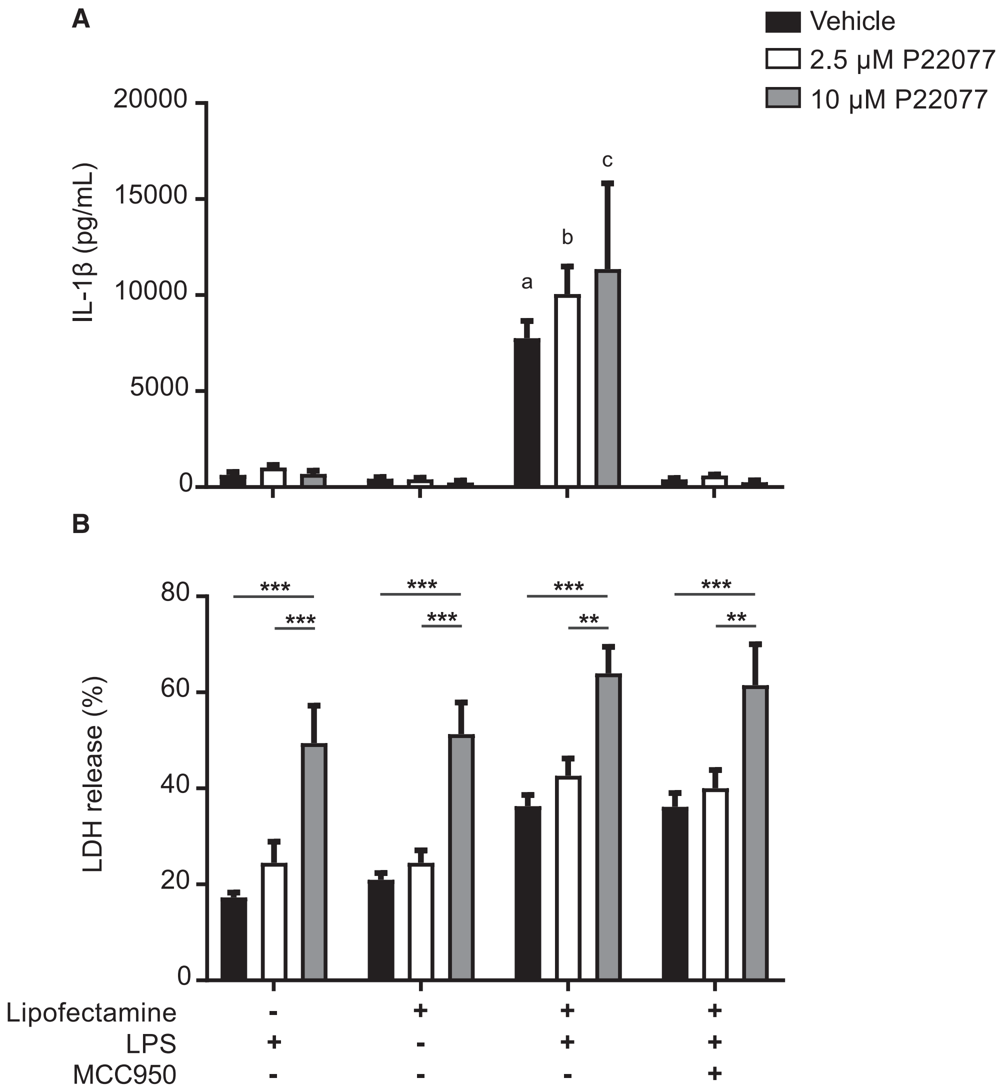

Figure EV4. Inhibition of USP7 and USP47 does not impair non-canonical inflammasome activation.

A IL-1β ELISA in supernatants of Pam3CSK4-primed (1 μg/ml, 4 h) murine BMDMs pre-incubated with either 0.1% DMSO (vehicle) or P22077 (2.5 μM and 10 μM, as indicated) for 15 min before transfection with LPS (2 μg/ml, 24 h). Bars represent the mean ± SD, n = 3 independent experiments. $^{a}P < 0.0001$; $^{b}P < 0.0001$ and $^{c}P < 0.0001$ when comparing Lipof./LPS versus LPS alone, Lipof. alone or LPS/Lipof./MCC950 within DMSO, P22077 2.5 μM or P222077 10 μM treatment correspondingly. No significant difference in IL-1β release in DMSO versus P22077 treatments within each group using two-way ANOVA.

B LDH release measured from cells treated as in (A). n = 3 independent experiments. $^{**}P < 0.01$ and $^{***}P < 0.001$ DMSO versus P22077 treatments within each group using two-way ANOVA.

© 2018 The Authors

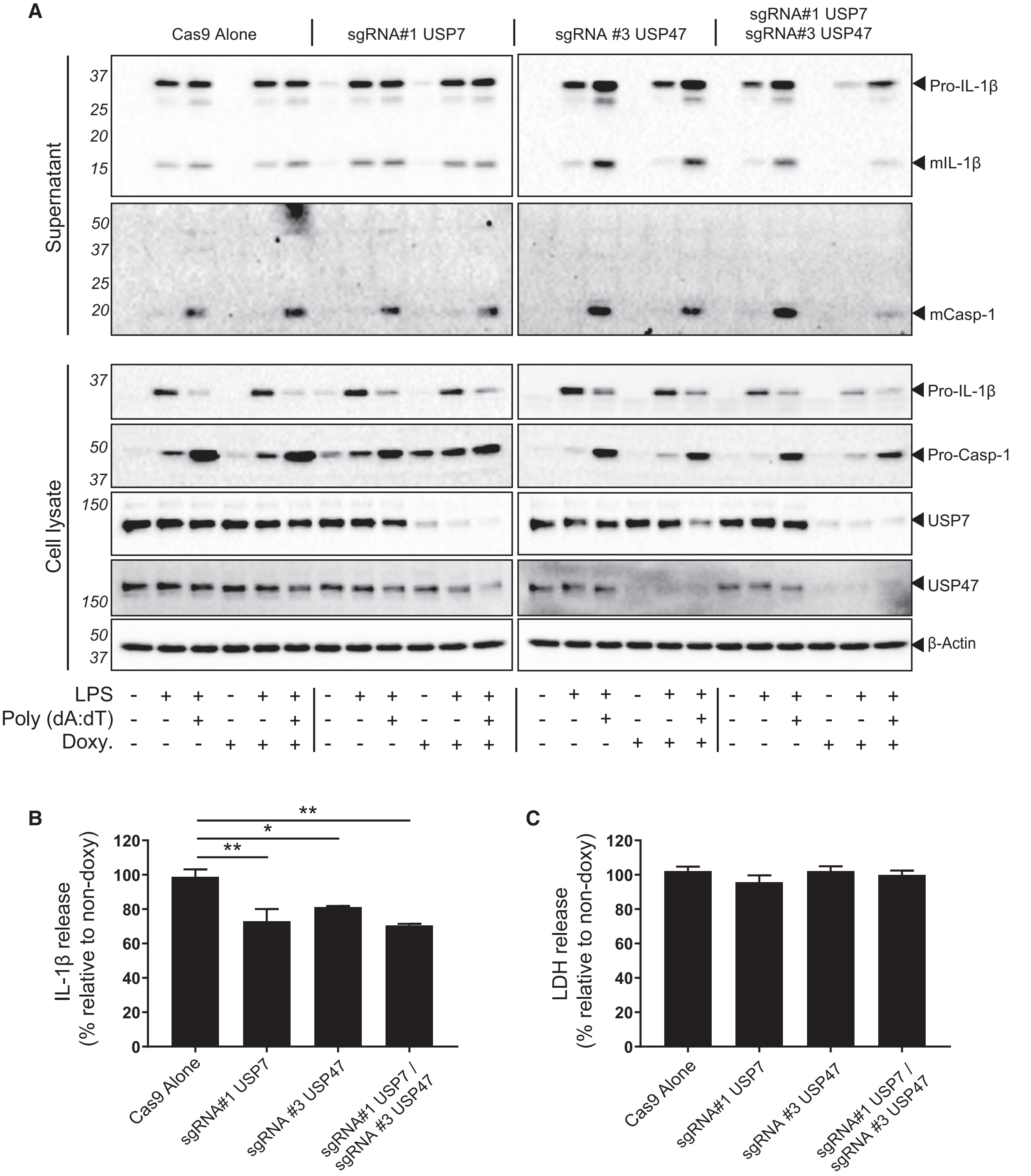
A
Cas9 Alone
sgRNA#1 USP7
sgRNA #3 USP47
sgRNA#1 USP7
sgRNA#3 USP47
Supernatant
Cell lysate
Pro-IL-1β
mIL-1β
mCasp-1
Pro-IL-1β
Pro-Casp-1
USP7
USP47
β-Actin
LPS
Poly (dA:dT)
Doxy.
B
IL-1β release
(% relative to non-doxy)
C
LDH release
(% relative to non-doxy)
Cas9 Alone
sgRNA#1 USP7
sgRNA #3 USP47
sgRNA#1 USP7 / sgRNA #3 USP47

Figure EV5.

Figure EV5. Inducible CRISPR/Cas9 knockout for USP7 and USP47 confirms their role in cGAS-STING-mediated NLRP3 inflammasome activation.

A Western blots of supernatants and cell lysates from PMA-differentiated THP-1 cells engineered by CRISPR/Cas9 to induce USP7 and/or USP47 KOs. Deficiency of both USP7 and USP47 was induced by doxycycline (Doxy) treatment (1 μg/ml; 3 days), as indicated. Cells were either unprimed or LPS-primed (1 μg/ml, 4 h) before Lipofectamine-based transfection with Poly (dA:dT) (5 μg/ml; 24 h), as indicated. Bands in the figure represent the following: pro-IL-1β (31 kDa); mature IL-1β (mIL-1β, 17 kDa); pro-caspase-1 (pro-Casp-1, 45 kDa); mature caspase-1 (mCasp-1, 20 kDa); and USP7 and USP47. β-Actin is shown as a loading control. Blots are representative of at least three independent experiments.

B IL-1β release measured from THP-1 cells treated as in (A), containing the indicated sgRNA. Bars represent the mean percentage of IL-1β release relative to their respective non-doxycycline-treated control cells ± SD, $n = 3$ independent biological replicates. *$P < 0.05$ and **$P < 0.01$ using a one-way ANOVA.

C LDH release measured from cells treated as in (A). Bars represent the mean percentage of LDH release relative to their respective non-doxycycline-treated control cells ± SD, $n = 4$ independent biological replicates. No statistical difference was found using one-way ANOVA.

Source data are available online for this figure.

© 2018 The Authors
