# Supplementary material for: USP7 and USP47 deubiquitinases regulate NLRP3 inflammasome activation
Source: EMBO Rep. 2018 Sep 11;19(10):e44766. doi: 10.15252/embr.201744766 (PMC6172458; doi:10.15252/embr.201744766)

Figure EV5. Raw Western blot images

A

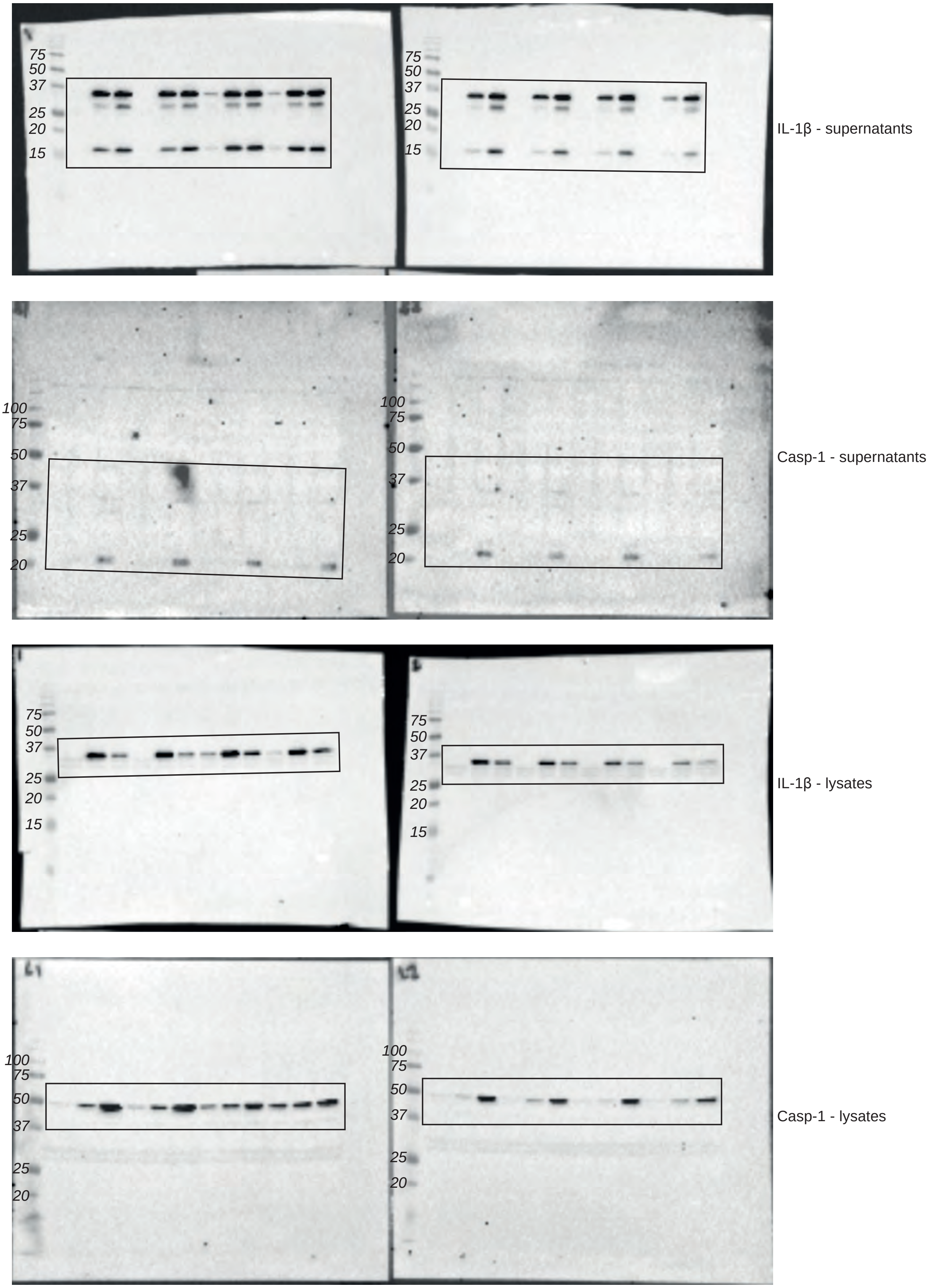

Figure EV5. Raw Western blot images continued...

A

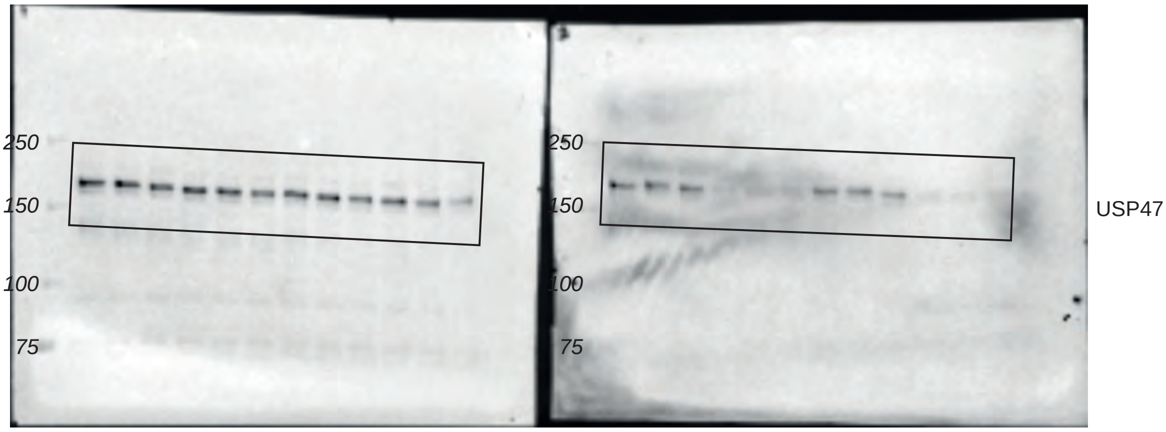

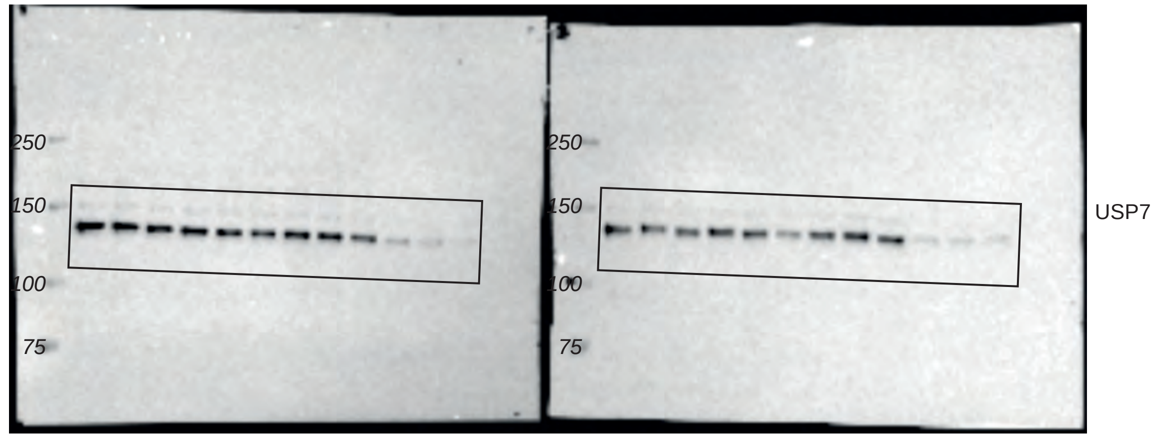

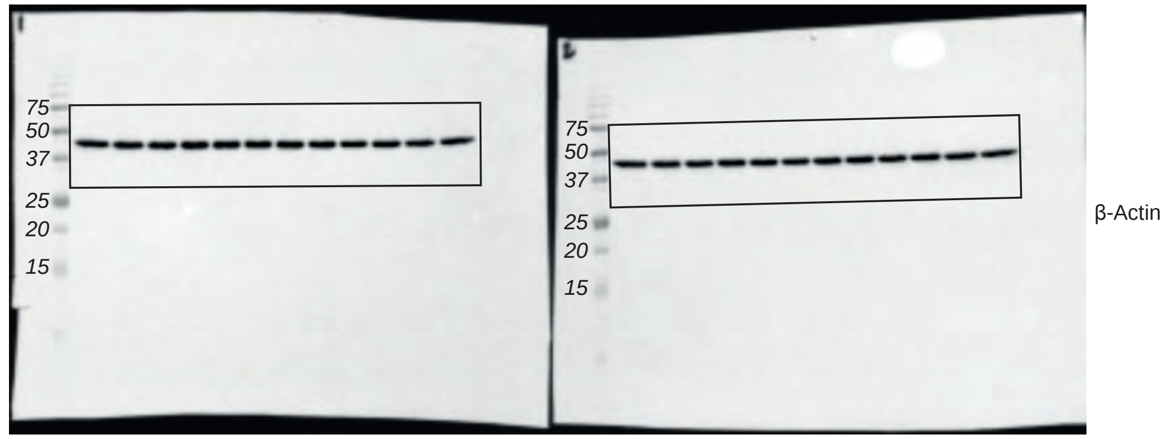

Supplement: Supplementary file 3 — Source Data for Expanded View and Appendix [file EMBR-19-e44766-s009.zip › Source_Data_for_EV_and_Appendix_Figures/Source_Data_for_FigEV5.pdf]
