# Supplementary figures and images for: USP7 and USP47 deubiquitinases regulate NLRP3 inflammasome activation
Source: EMBO Rep. 2018 Sep 11;19(10):e44766. doi: 10.15252/embr.201744766 (PMC6172458; doi:10.15252/embr.201744766)

Figure EV1. Raw Western blot images

B

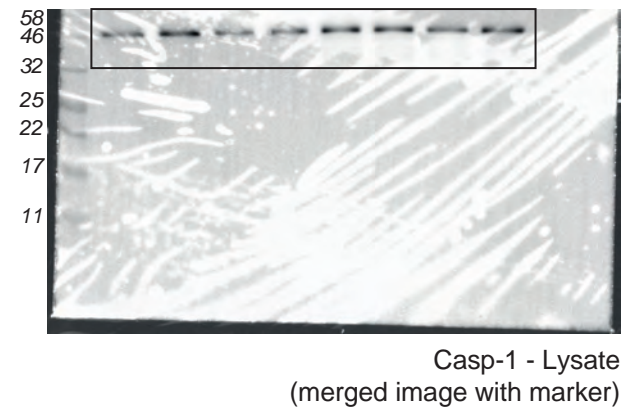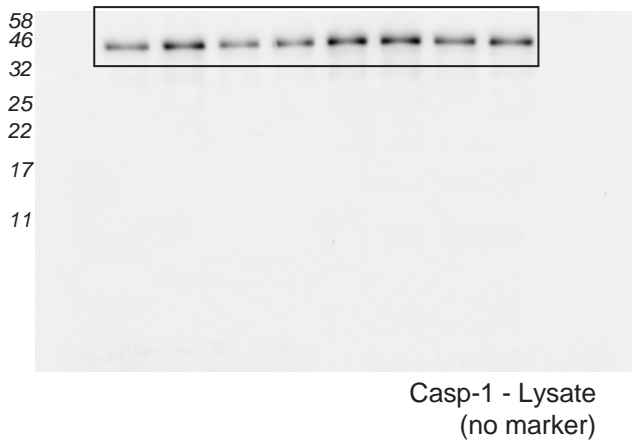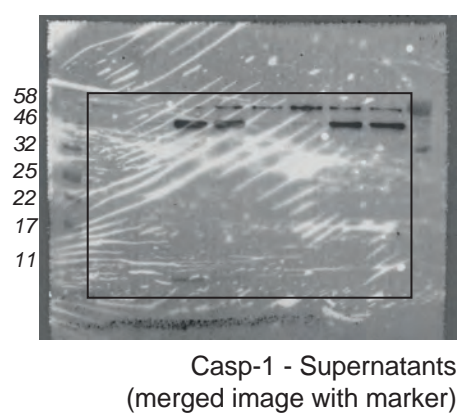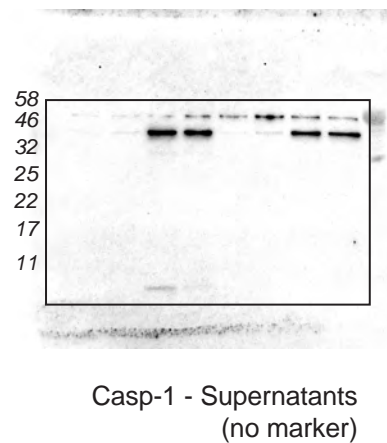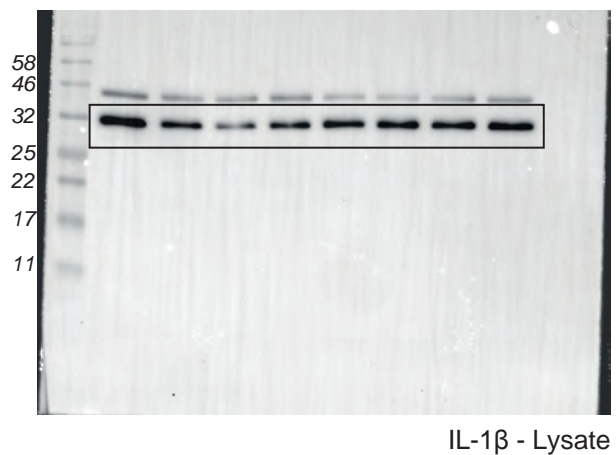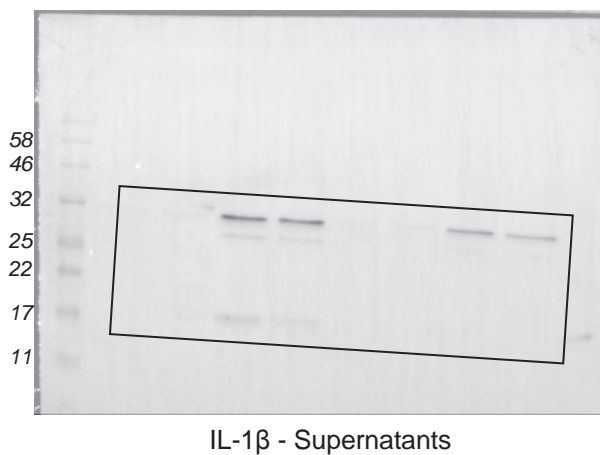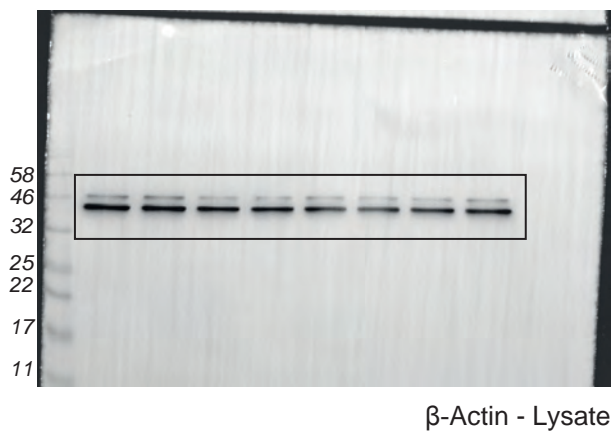

Supplement: Supplementary file 3 — Source Data for Expanded View and Appendix [file EMBR-19-e44766-s009.zip › Source_Data_for_EV_and_Appendix_Figures/Source_Data_for_FigEV1.pdf]

Figure EV2. Raw Western blot images

C

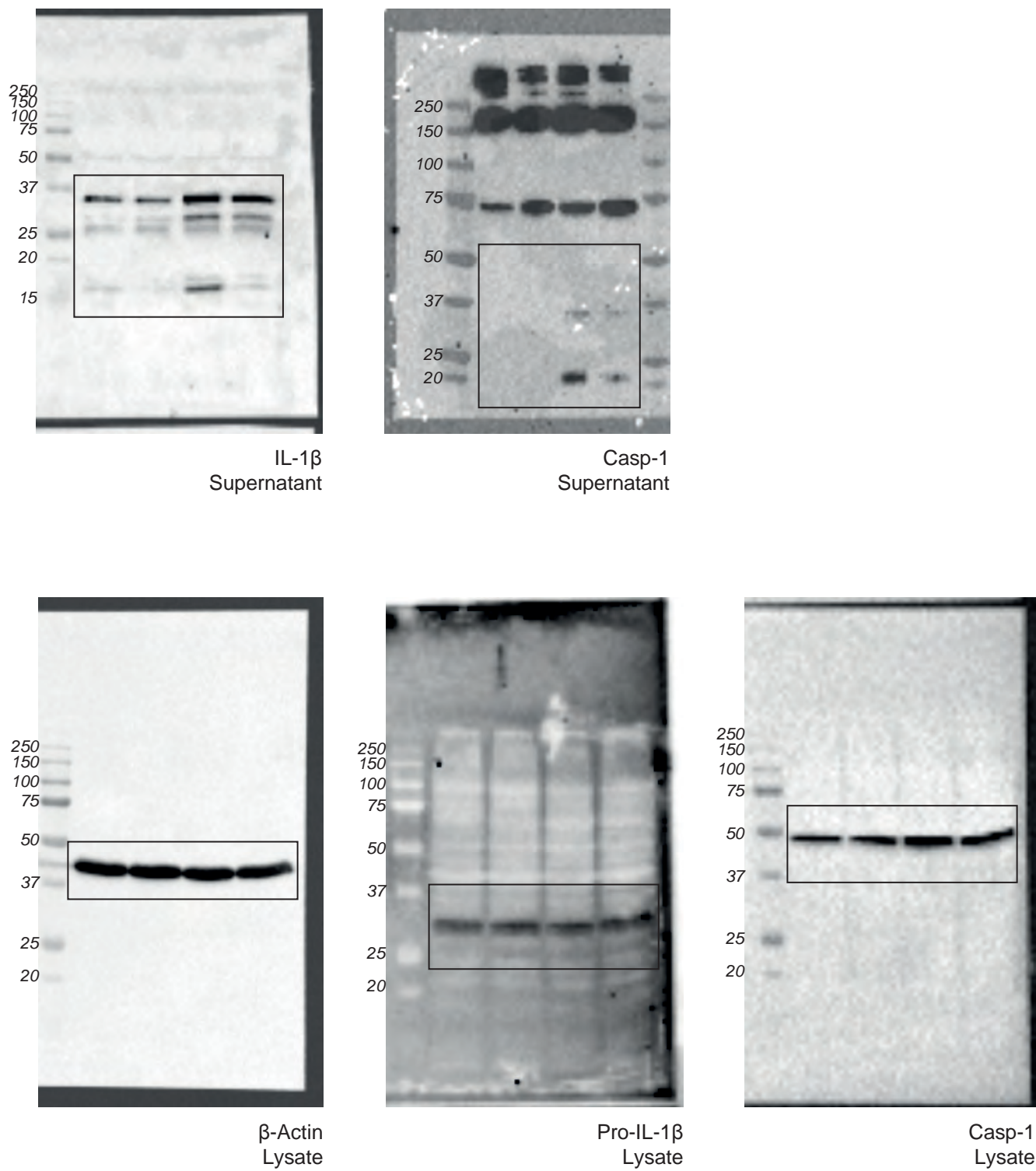

Supplement: Supplementary file 3 — Source Data for Expanded View and Appendix [file EMBR-19-e44766-s009.zip › Source_Data_for_EV_and_Appendix_Figures/Source_Data_for_FigEV2.pdf]

Figure EV3. Raw Western blot images

C

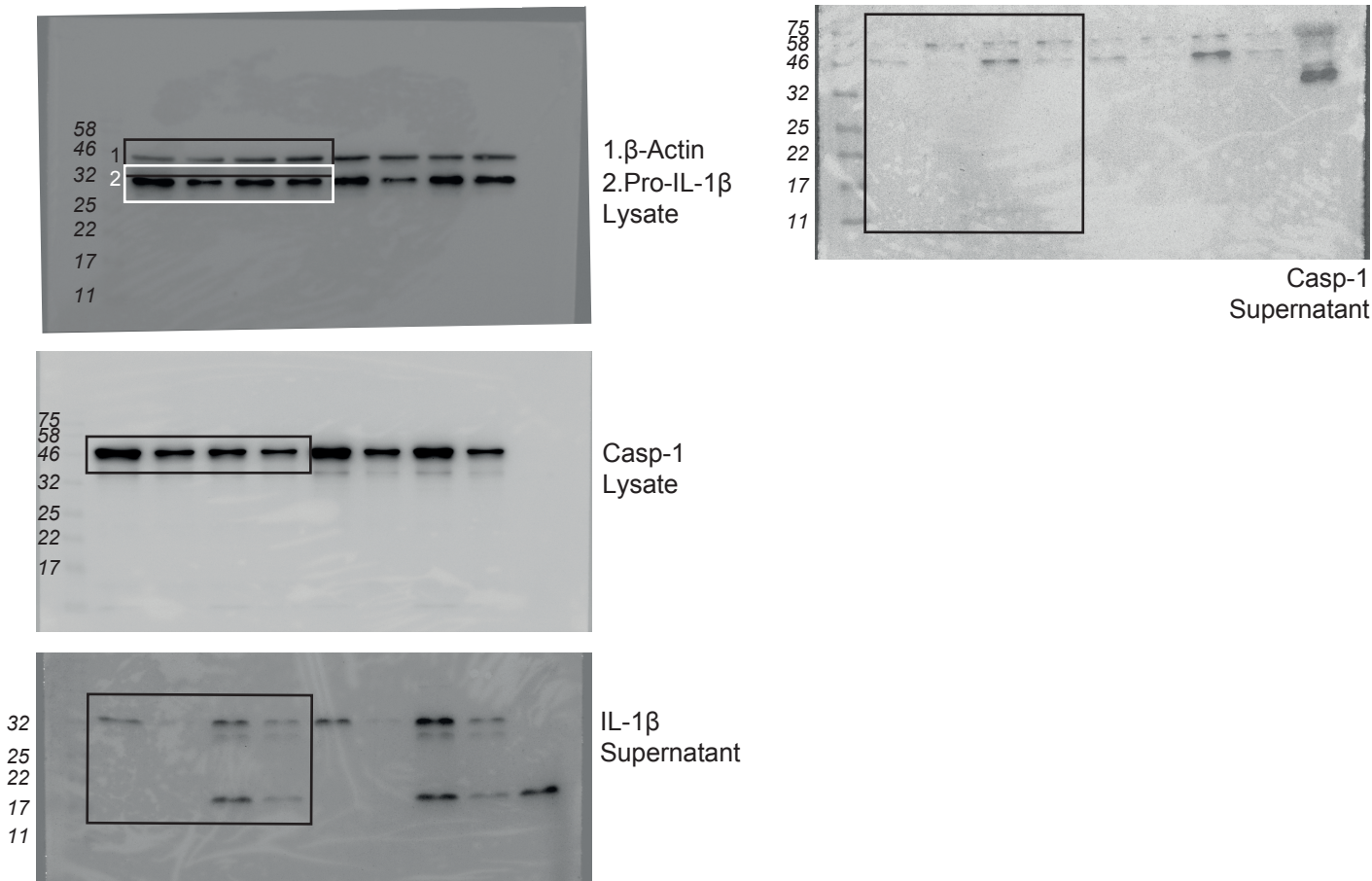

D

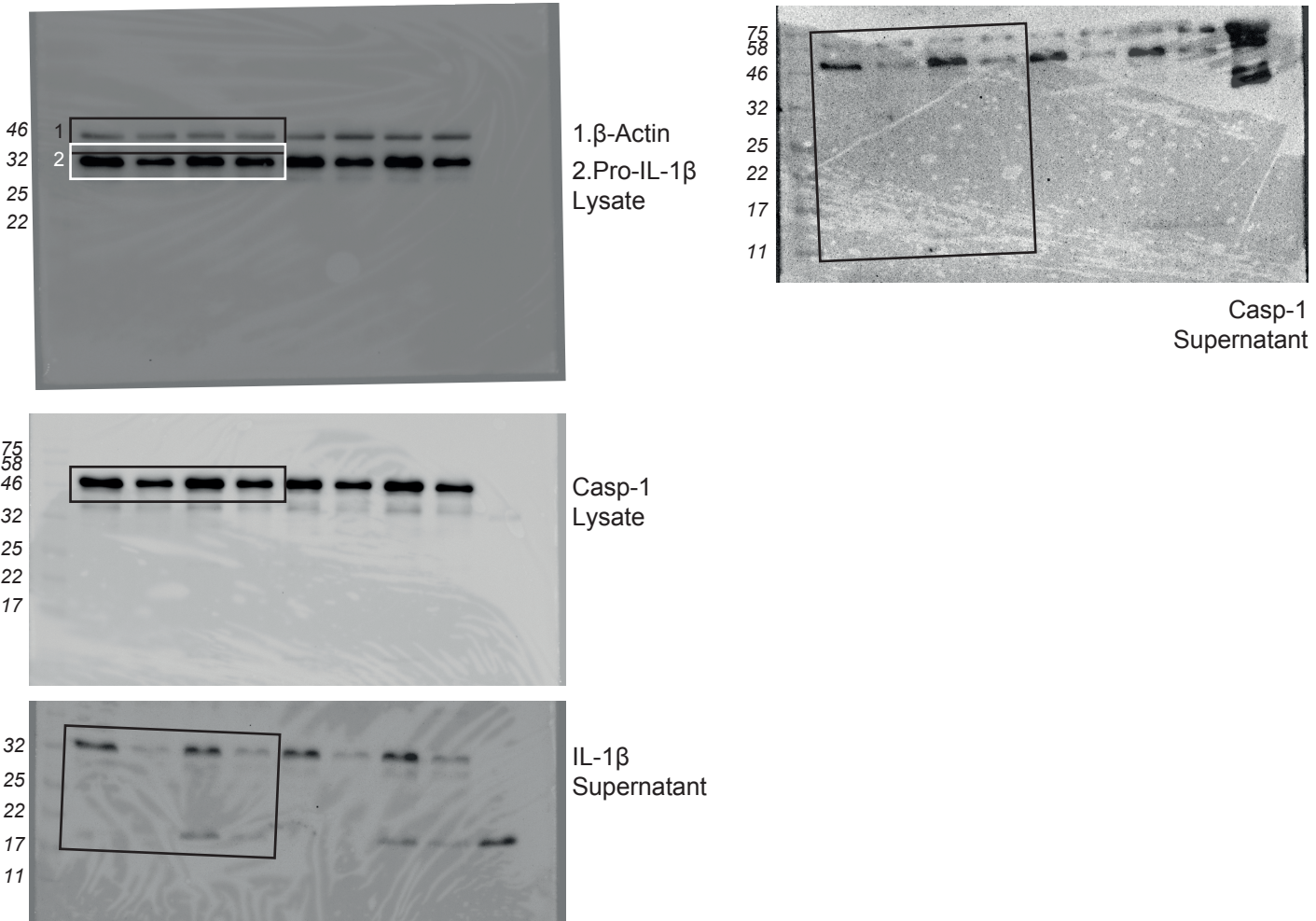

Supplement: Supplementary file 3 — Source Data for Expanded View and Appendix [file EMBR-19-e44766-s009.zip › Source_Data_for_EV_and_Appendix_Figures/Source_Data_for_FigEV3.pdf]

Figure 2. Raw Western blot images

B.

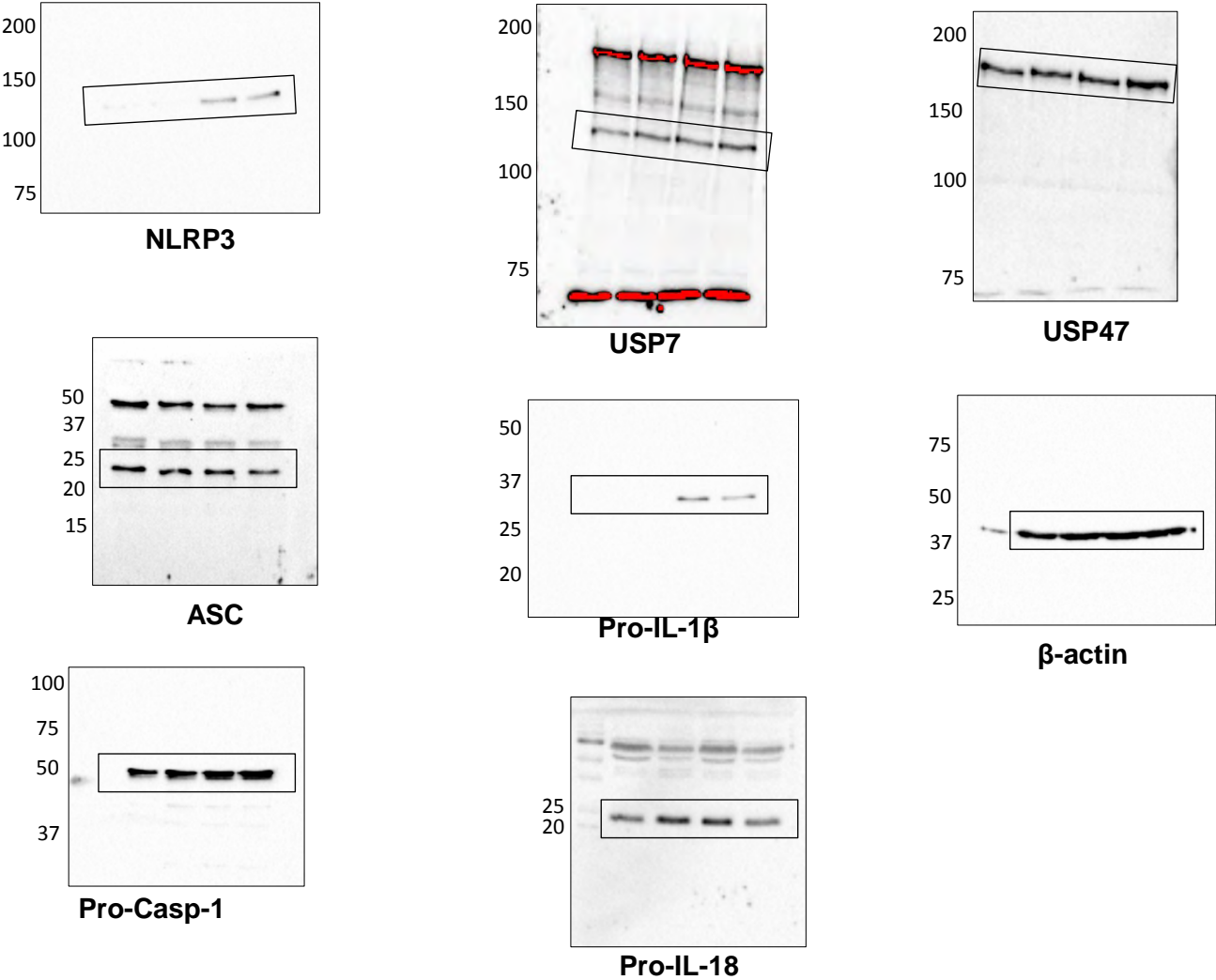

D.

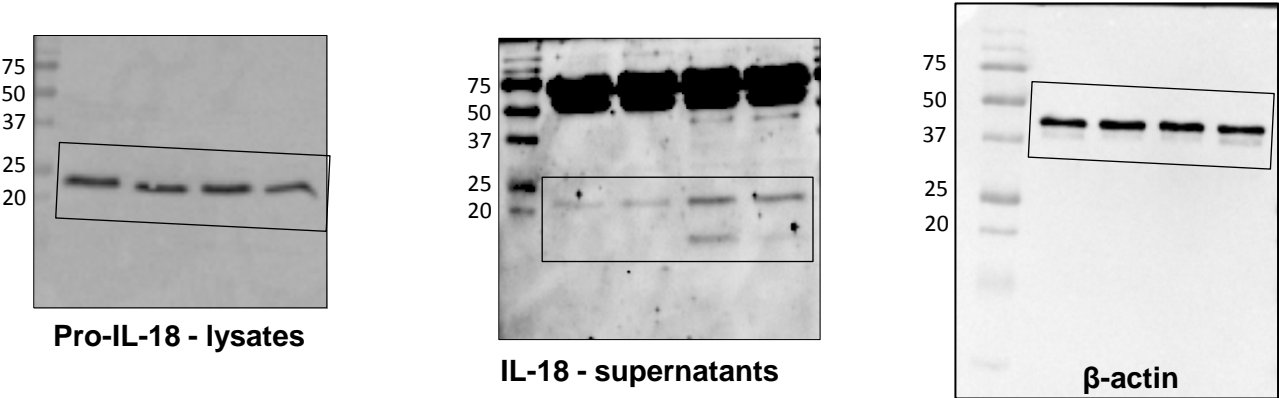

Supplement: Supplementary file 6 — Source Data for Figure 2 [file EMBR-19-e44766-s004.pdf]

Figure 3. Raw Western blot images

A

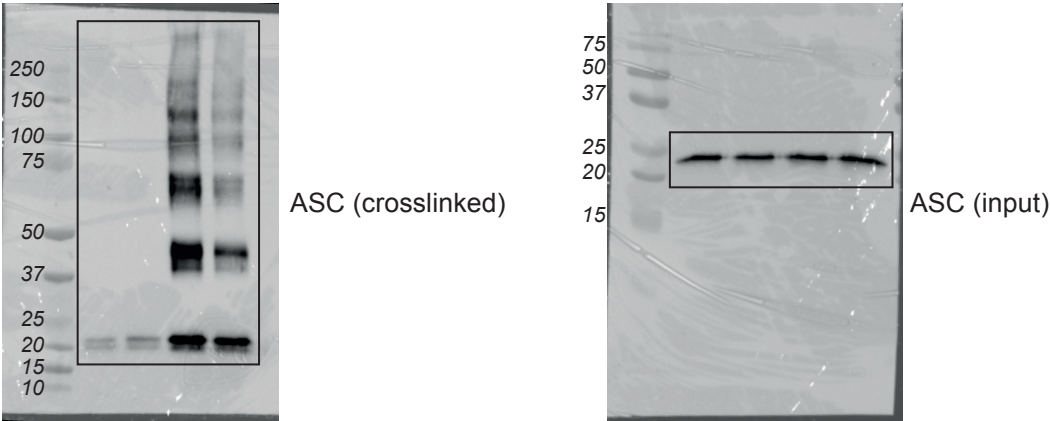

B

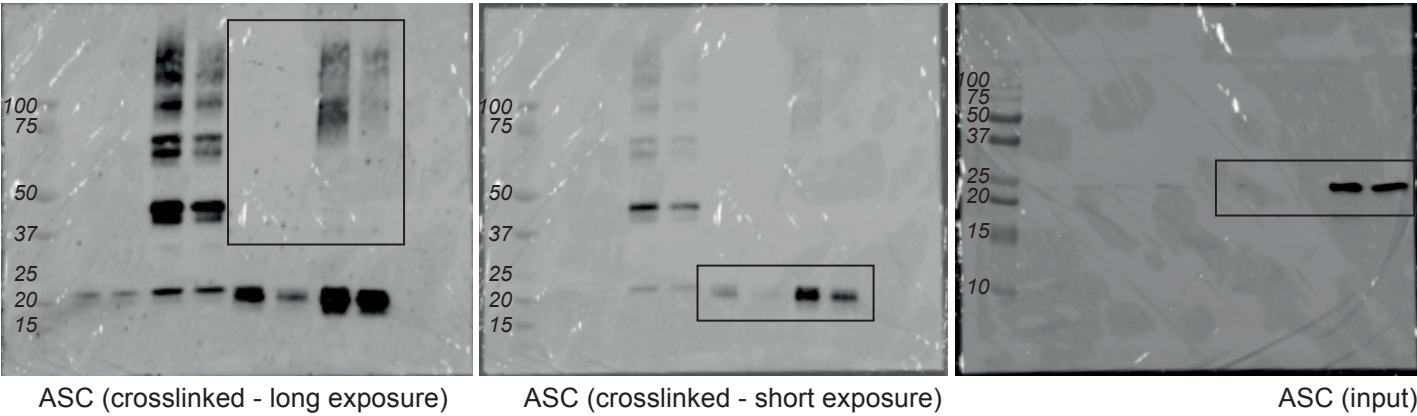

Supplement: Supplementary file 7 — Source Data for Figure 3 [file EMBR-19-e44766-s005.pdf]

Figure 5. Raw Western blot images

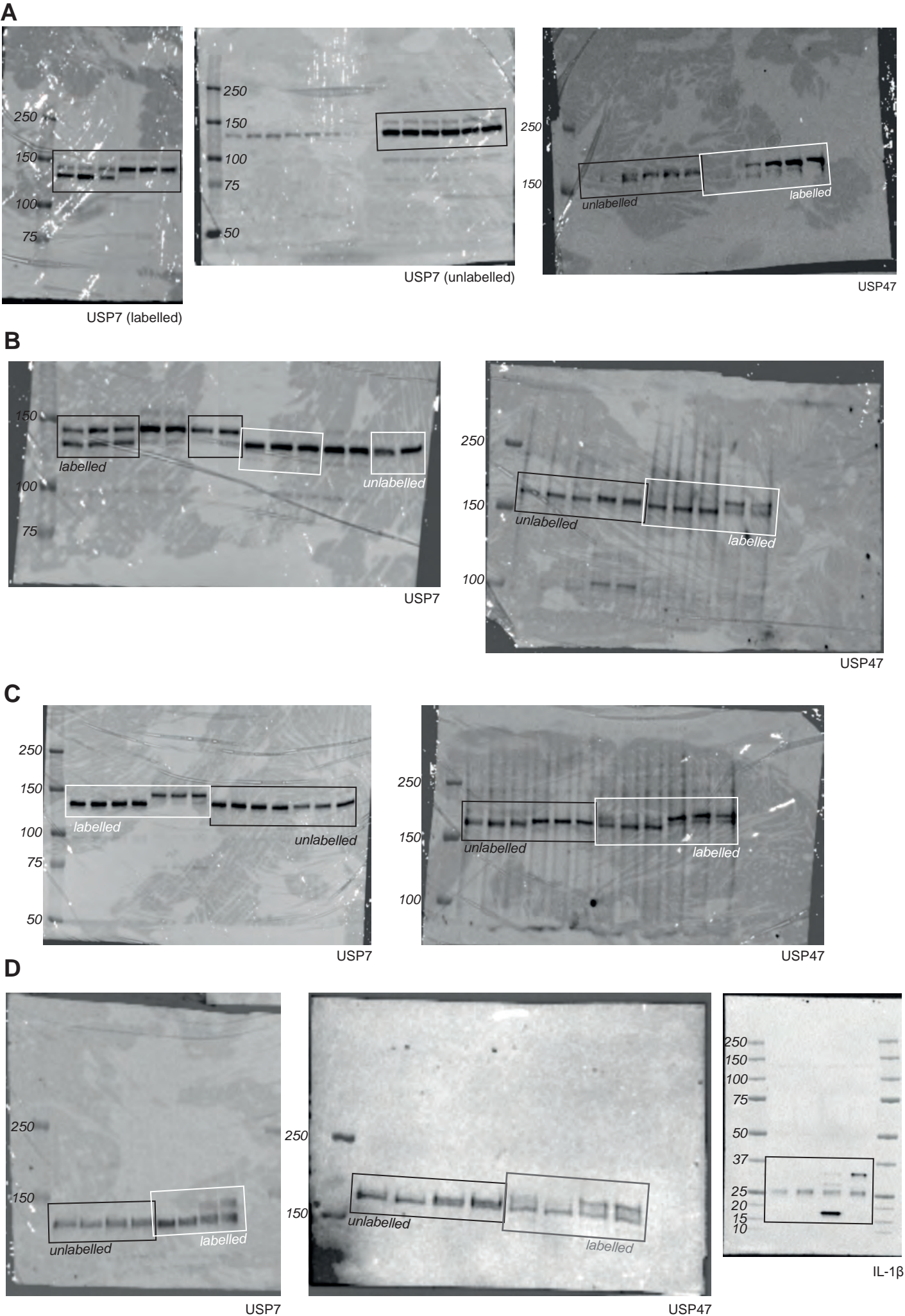

Supplement: Supplementary file 9 — Source Data for Figure 5 [file EMBR-19-e44766-s007.pdf]

Fig 6. Raw Western blot images - supernatants

A

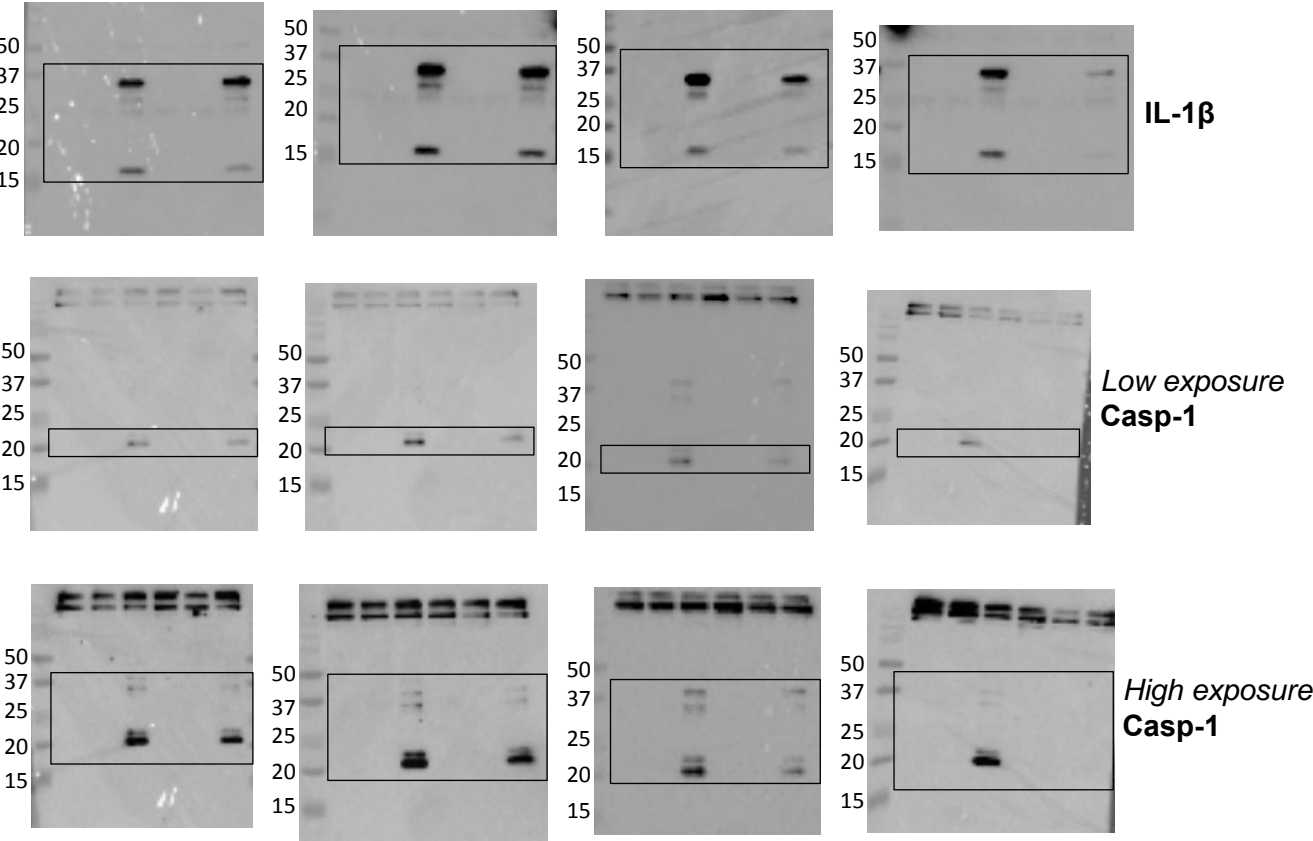

**Fig 6. Raw Western blot images - lysates**

**A**

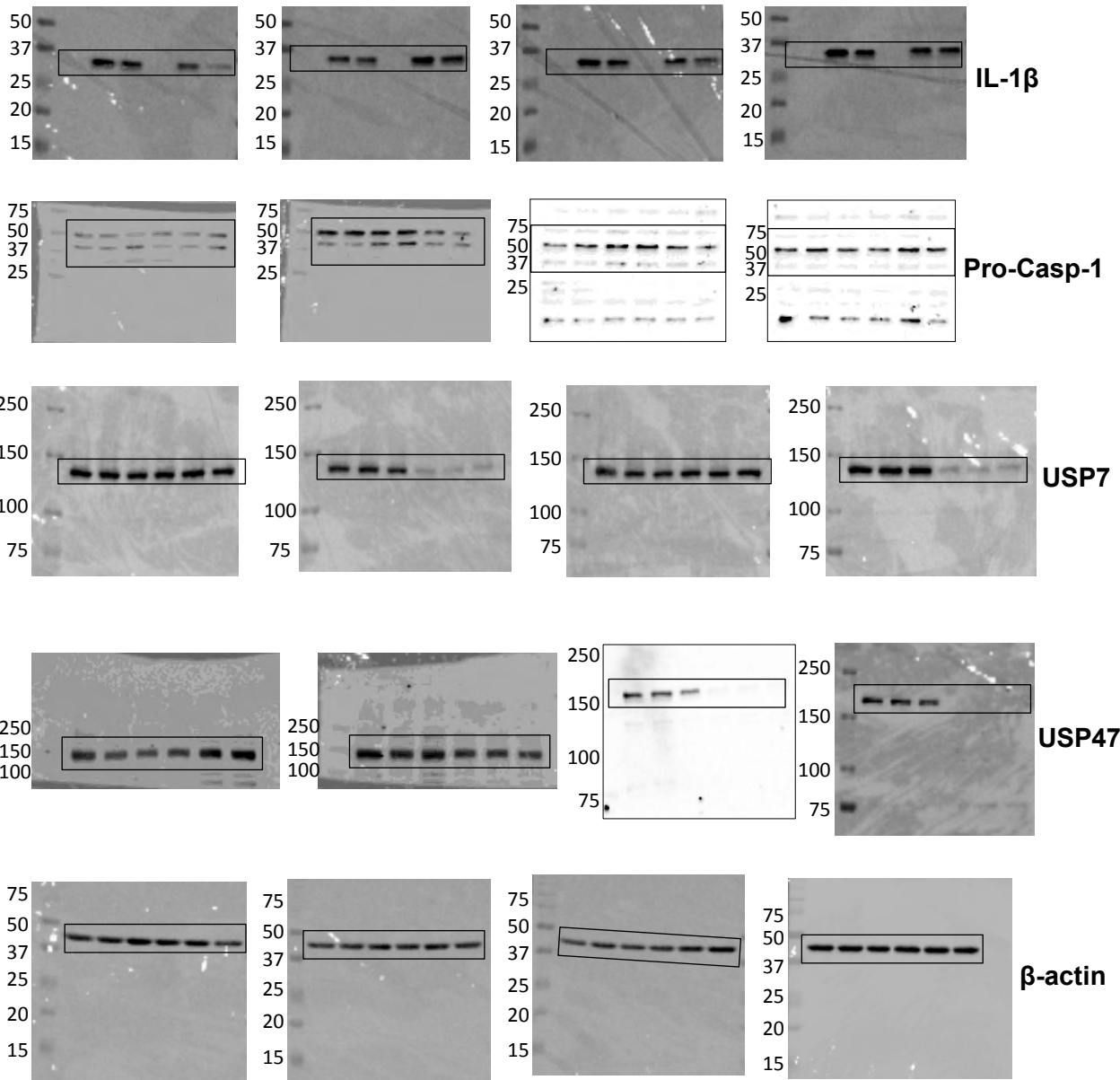

Supplement: Supplementary file 10 — Source Data for Figure 6 [file EMBR-19-e44766-s008.pdf]
