# Supplementary material for: USP7 and USP47 deubiquitinases regulate NLRP3 inflammasome activation
Source: EMBO Rep. 2018 Sep 11;19(10):e44766. doi: 10.15252/embr.201744766 (PMC6172458; doi:10.15252/embr.201744766)
Supplement: Supplementary file 5 — Source Data for Figure 1 [file EMBR-19-e44766-s003.pdf]

Figure 1. Raw Western blot images
D

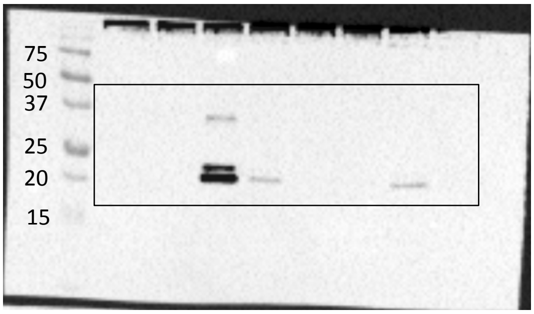

Short exposure
Caspase-1 - supernatant

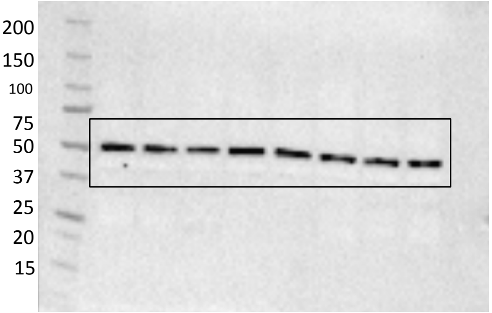

Caspase-1 - lysate

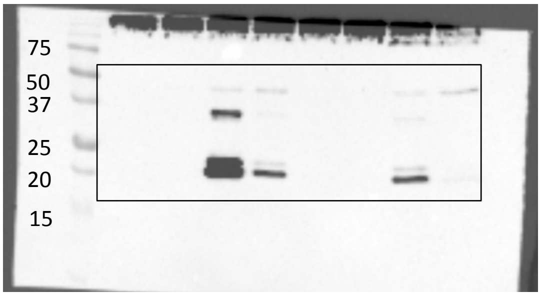

Long exposure
Caspase-1 - supernatant

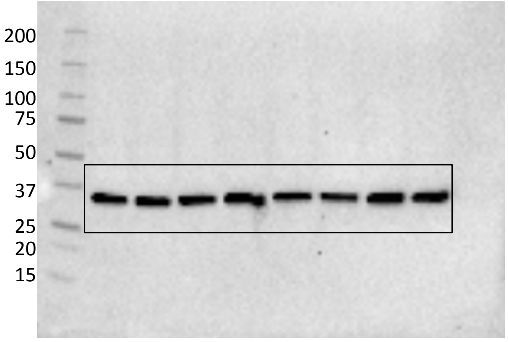

IL-1β - lysate

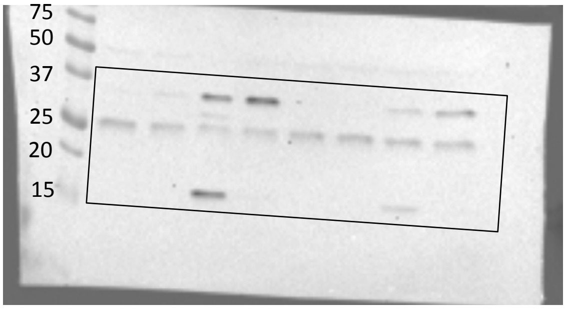

IL-1β - supernatant

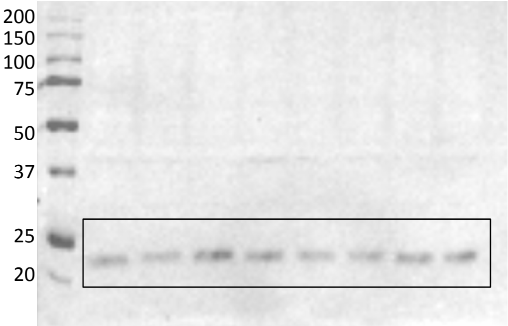

IL-18 – lysate

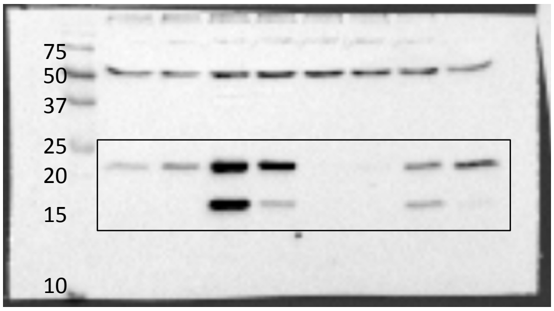

IL-18 Supernatant

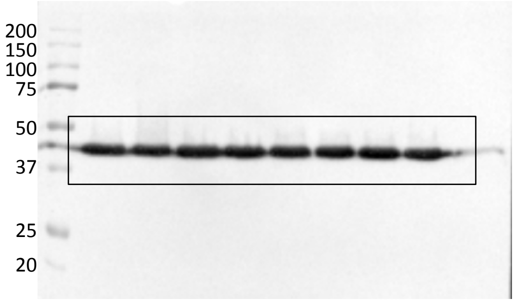

β-actin - Lysate
