# Supplementary material for: USP7 and USP47 deubiquitinases regulate NLRP3 inflammasome activation
Source: EMBO Rep. 2018 Sep 11;19(10):e44766. doi: 10.15252/embr.201744766 (PMC6172458; doi:10.15252/embr.201744766)

Fig 4A. Western blot RAW images

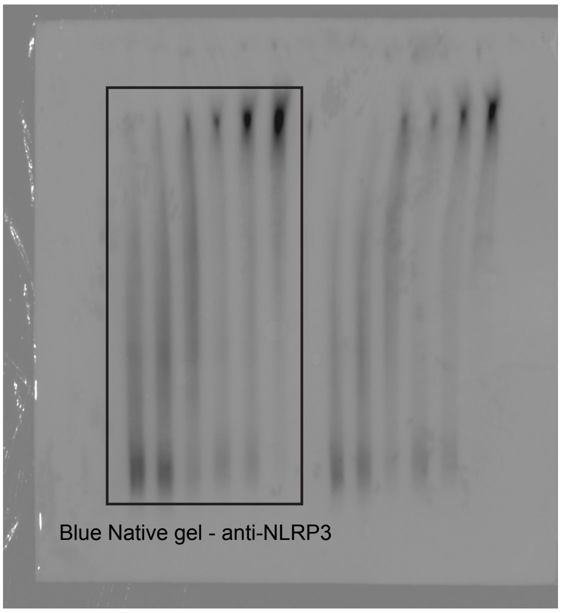

Blue Native gel - anti-NLRP3

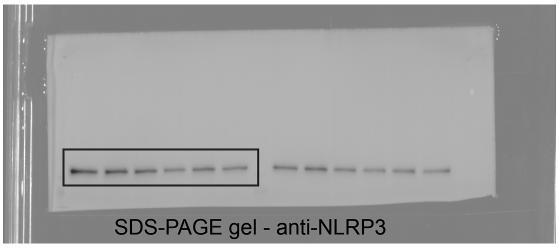

SDS-PAGE gel - anti-NLRP3

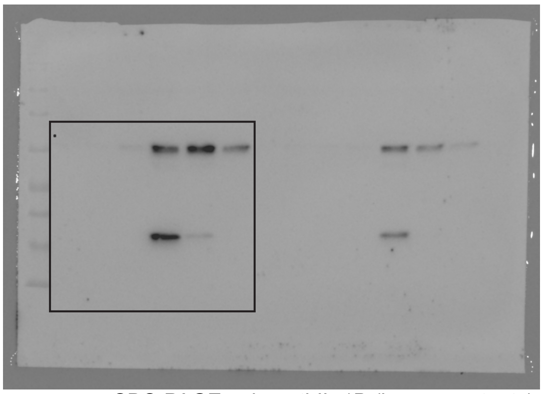

SDS-PAGE gel - anti-IL-1B (in supernatants)

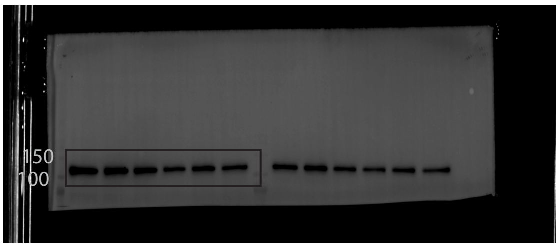


SDS-PAGE gel - anti-NLRP3
(Adjusted contrast so you can observe the ladder)

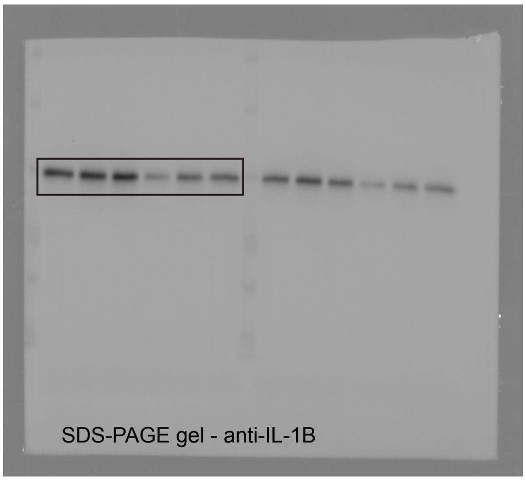

SDS-PAGE gel - anti-IL-1B

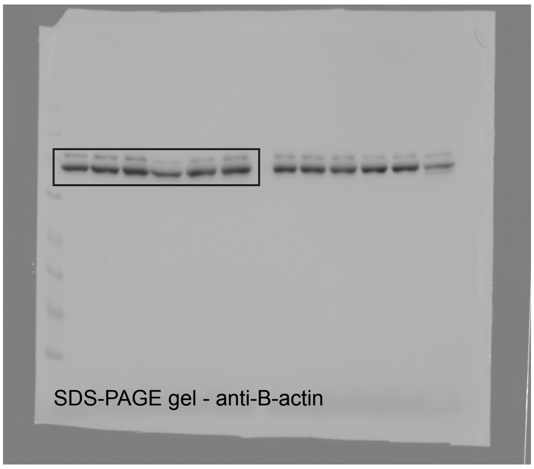

SDS-PAGE gel - anti-B-actin

Fig 4B. RAW Western Blot images

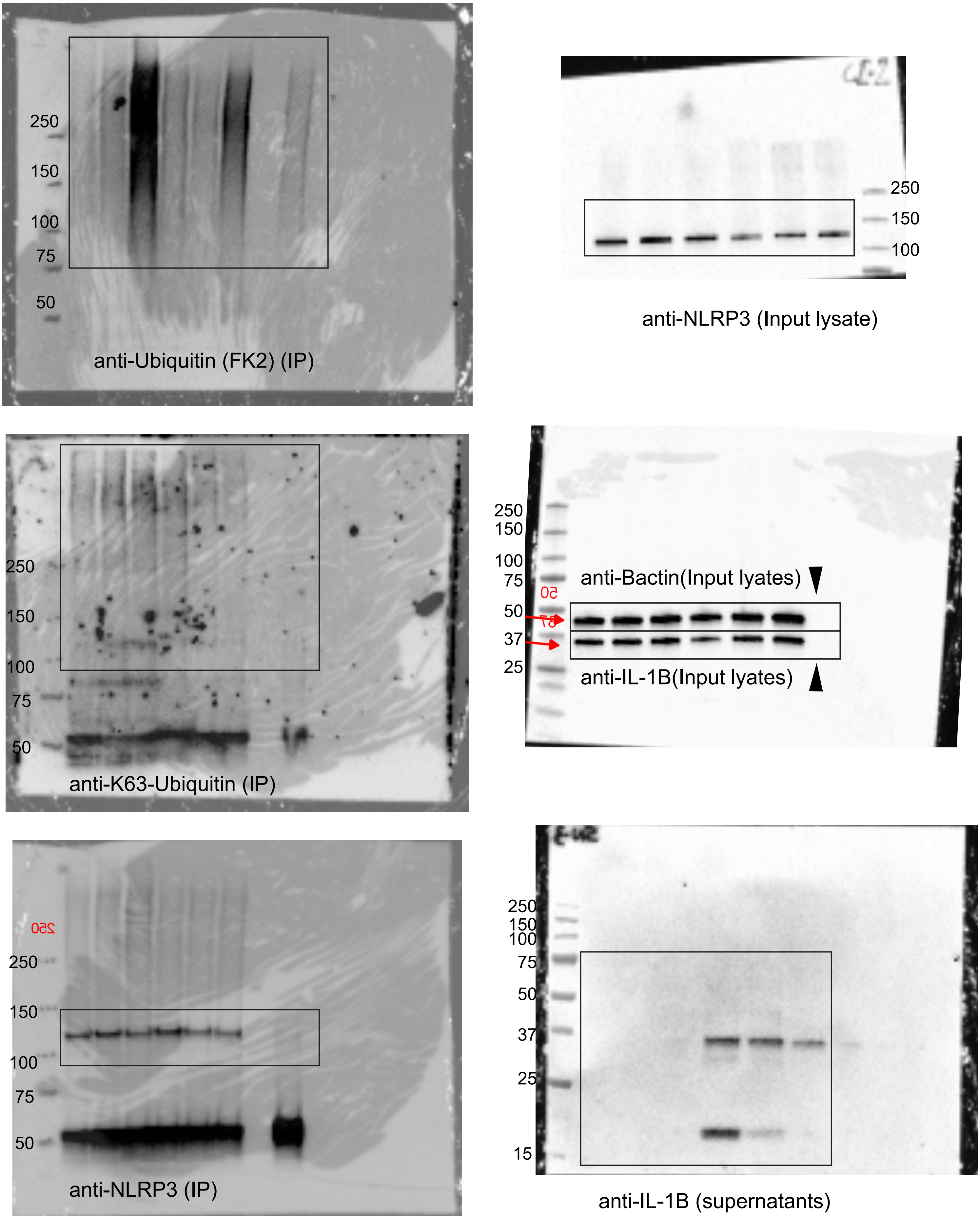

Supplement: Supplementary file 8 — Source Data for Figure 4 [file EMBR-19-e44766-s006.pdf]
